# A Mathematical Model of Cellular Aggregation Predicts Patterns of Tau Accumulation in Neurodegenerative Disease

**DOI:** 10.1002/advs.202511297

**Published:** 2025-10-27

**Authors:** Shih‐Huan Huang, Annelies Quaegebeur, Tanrada Pansuwan, Timothy Rittman, Ruiyan Wang, Tuomas PJ Knowles, James B Rowe, David Klenerman, Georg Meisl

**Affiliations:** ^1^ Yusuf Hamied Department of Chemistry University of Cambridge Cambridge CB2 1EW UK; ^2^ Department of Clinical Neurosciences and Cambridge University Hospitals NHS Trust University of Cambridge Cambridge CB2 0SZ UK; ^3^ UK Dementia Research Institute University of Cambridge Cambridge CB2 0AH UK

**Keywords:** mechanistic model, molecular mechanism, neurodegeneration, protein aggregation, tauopathy

## Abstract

Protein aggregates are a hallmark of neurodegenerative disease, yet the molecular processes that control their appearance remain incompletely characterized. In particular, it is unknown to what degree the development of aggregates in one cell is triggered by nearby aggregate‐containing cells, as opposed to proceeding cell‐autonomously. Here, a minimal, bottom‐up computational model is developed that is characterized by just two parameters: the relative rate of cell autonomous and cell‐to‐cell triggers of aggregation and a length scale of cell‐to‐cell interactions. Its applicability is demonstrated in the primary tauopathy Progressive Supranuclear Palsy by extracting mechanistic information from the distribution of tau aggregates at different disease stages from post‐mortem human brain. Despite its simplicity, the model is able to reproduce the aggregate patterns observed in the data and reveals that the triggering of aggregation by nearby aggregated cells, over distances of ≈100 µm, is the major driver of disease progression once a low threshold level of aggregates is reached. The model also provides a natural explanation for an increase in the rate of disease progression when this threshold is reached, providing fundamental new insights into disease mechanisms and predicting the efficiency of different therapeutic strategies.

## Graphical abstract

A minimal computational model of cellular aggregate formation in neurodegenerative disease, encompassing two competing processes: cell‐autonomous and cell‐to‐cell triggers of aggregation, is presented. Despite its simplicity, the model can reproduce the aggregate patterns observed in post‐mortem brain images from the primary tauopathy Progressive Supranuclear Palsy, identifying the triggering of aggregation by already aggregated cells as the major driver of progression.

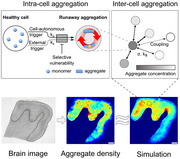

## 1 Introduction

The association between misfolded, aggregating proteins and neurodegenerative diseases is well established.^[^ ^1^, ^2^ ^]^ Even though aggregating proteins are diverse and the aggregated structures they give rise to are specific to each diseasen,^[^ ^3^ ^]^ there are common principles of pathogenesis at the molecular and cellular level. These principles include the intrinsic ability of aggregating protein species to self‐replicate,^[^ ^4^ ^]^ their ability to overwhelm or avoid protein quality control and removal mechanisms,^[^ ^5^, ^6^, ^7^ ^]^ and the potential to spread between cells.^[^ ^8^, ^9^, ^10^ ^]^ It has been shown that both aggregate self‐replication and spreading occur in cell and animal models.^[^ ^11^, ^12^, ^13^ ^]^ However, in most cases it has yet to be shown for human neurodegenerative diseases which is the critical – or rate‐limiting – process. Determining this process is essential to guide therapeutic strategies to slow or arrest disease progression. Therefore, a quantitative model of the molecular drivers of disease, that works at the spatio‐temporal scale of human disease, is required.^[^ ^14^ ^]^

Protein aggregation plays a central role across a spectrum of neurodegenerative diseases and has been studied in detail in vitro.^[^ ^4^, ^15^, ^16^ ^]^ Yet, the cellular and molecular processes found to control aggregate formation under such controlled conditions have proven difficult to relate to disease emergence or the pattern and pace of pathology observed in human disease.^[^ ^17^ ^]^ Mathematical modeling of disease progression can bridge this gap, to link macroscopic patterns of progression to cellular and molecular processes. For example, models of selective vulnerability and connectivity can recover the brain‐wide patterns of regions becoming affected in sequence over the course of different diseases.^[^ ^18^ ^]^ Using such models, the rates of general classes of pathological processes have been quantified,^[^ ^12^ ^]^ and the interaction of beta‐amyloid and tau aggregation in Alzheimer's disease (AD) has been elucidated.^[^ ^19^ ^]^

To date, such models of human disease have focused mainly on macro‐scale modeling, informed by whole‐brain imaging methods such as positron emission tomography (PET), magnetoencephalography (MEG) and magnetic resonance imaging (MRI).^[^ ^20^, ^21^, ^22^ ^]^ The resolution of these techniques limits the conclusions that can be drawn about molecular and cellular mechanisms, and the applicability in drug development, which by its nature acts at the molecular level. New modeling approaches are therefore required that accommodate micro‐ and meso‐scale processes. With the advance of digital pathology and AI‐facilitated automated classification of aggregated cell types,^[^ ^23^ ^]^ cellular‐level resolution of neuropathological changes can now be obtained and quantified at scale.^[^ ^23^, ^24^ ^]^ Such digital data with cellular resolution opens the door for models to investigate cell‐level aggregation drivers and cell‐to‐cell interactions.

Here, we present a model to link cellular aggregation patterns observed at the tissue level to underlying cellular processes. To achieve this, we use a minimal model of aggregation in a cell, and then allow cells to interact with each other to trigger aggregation. We apply this model to data from the primary tauopathy Progressive Supranuclear Palsy (PSP). PSP is associated with the aggregation of misfolded 4‐repeat (4R) tau,^[^ ^25^ ^]^ independent of the aggregation of a second misfolded protein (e.g. amyloid‐beta in Alzheimer's disease). PSP therefore provides an ideal test‐bed for our approach, with its high clinico‐pathological correlation, and the high propensity of its 4R‐tau to aggregate. Moreover, tau pathology in PSP follows a stereotypical sequential pattern, as described for PSP‐Richardson's syndrome by Kovacs et al.^[^ ^26^ ^]^

We first introduce the mathematical model and demonstrate its behavior when the different processes dominate. We then use an automated image analysis approach to capture the spatial aggregate patterns of histopathological images from 12 brain regions from 11 brain donors with PSP, across disease stages^[^ ^26^ ^]^ (details see Table S1, Supporting Information). The digitized information from nuclei and pathological tau deposits is then used in our model to determine the relative contribution of the individual processes that gave rise to the observed patterns. This allows us to determine the rate‐limiting steps in the appearance of pathological aggregates and the progression of disease, and investigate how the contribution of cell‐to‐cell and cell‐autonomous processes varies across brain regions and over the course of the disease. Our model matches experimentally observed patterns and naturally yields a switch between a slower, cell‐autonomous phase and a faster cell‐to‐cell interaction‐dominated phase of the disease, explaining an observed increase in the rates of progression as disease advances. We anticipate that lessons learned here will also inform models of AD and other aggregation‐related neurodegenerative diseases.

## 2 Results

### 2.1 Modeling Cell‐Level Aggregate Formation

A number of processes are needed to describe cellular‐level patterns of aggregate formation in neurodegenerative diseases such as PSP. The key processes of aggregate formation are: i) *initiation*, de‐novo formation of aggregates without the involvement of existing aggregates; ii) *multiplication*, formation of new aggregates triggered by existing aggregates, for example, via fragmentation; and iii) *growth*, growth of existing aggregates by addition of further proteins. These processes couple together into a minimal reaction network that produces auto‐catalytic amplification of aggregates, a feature observed for tau and across disease‐associated proteins.^[^ ^4^, ^27^ ^]^ Protein synthesis and aggregate removal also play important roles in cellular aggregation, resulting in two distinct states for a cell: stable (healthy) and runaway aggregation (diseased).^[^ ^5^, ^28^ ^]^

In our model in silico system, we included the following core features: cells are either in a stable state or a runaway aggregation state. They remain in the stable state unless triggered to switch to the runaway aggregation state, either by a random cell‐autonomous event, or by influence from other cells in the runaway aggregation state. Once triggered, a cell quickly accumulates aggregates.^[^ ^28^, ^29^ ^]^ In a real system, this switch can occur when aggregate self‐replication overwhelms clearance, or when significant amounts of preformed seed enter the cell.^[^ ^5^ ^]^ We assume that the large aggregate deposits visible in histological stains occur only in cells that have switched to the runaway aggregation state, and we refer to these as *aggregated cells* throughout.

**Figure 1:**
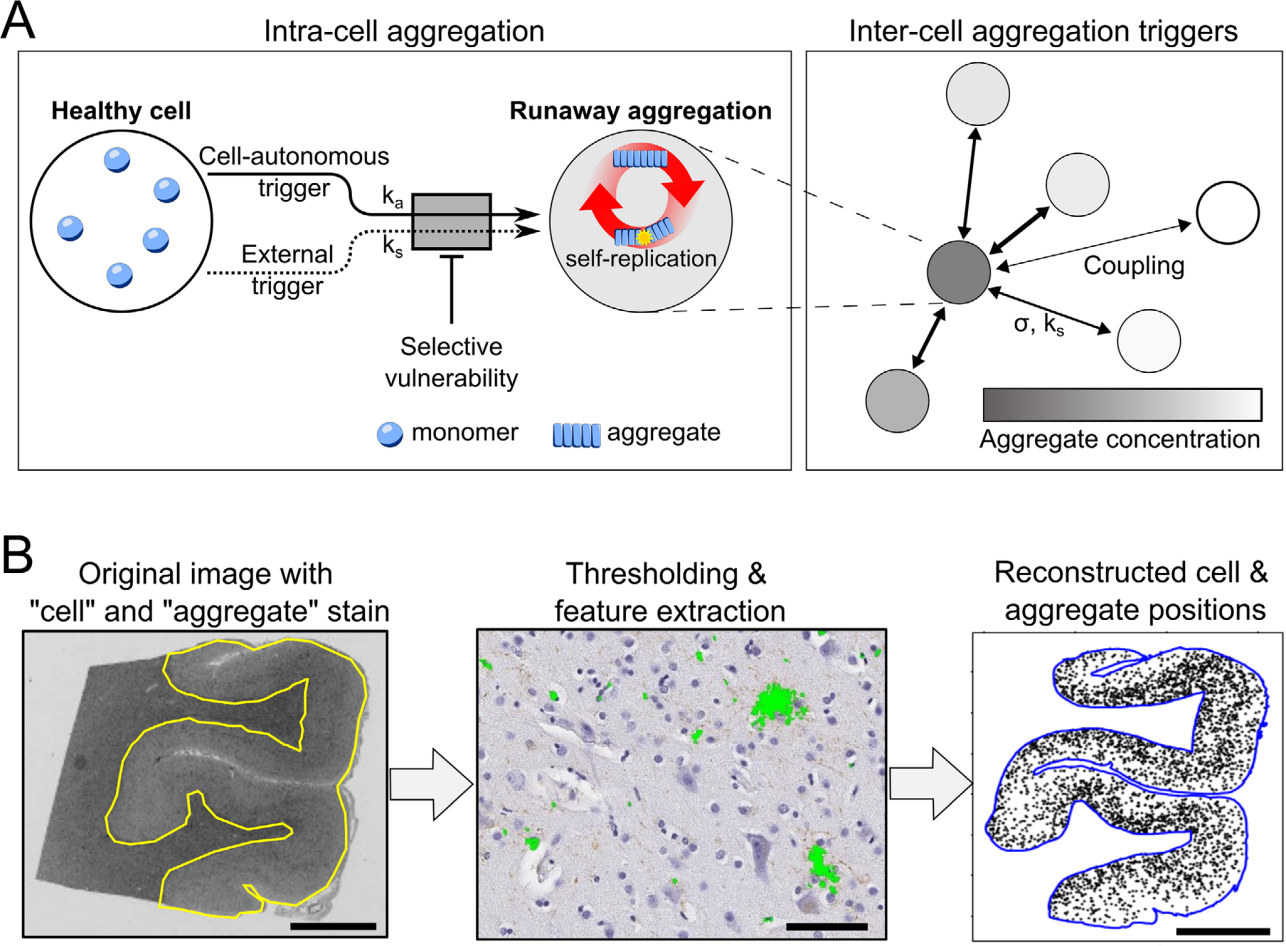
Schematic of the in vivo cellular model for protein aggregation and our data processing pipeline. A) In our model, the basic unit is a cell. Within each cell, once aggregation is triggered, aggregates rapidly accumulate. Aggregation can be triggered cell‐autonomously (rate *k*_*a*_, units of inverse time), and additionally aggregated cells can trigger aggregation in other cells (as a model for e.g. transfer of seeds or indirectly mediated triggers), with a length‐dependent coupling strength between cells (rate *k*_*s*_ in units of inverse time, length dependence σ in units of length). How susceptible to being triggered a cell is, is determined by its *vulnerability*. As the simulation proceeds, we track the aggregation state of each cell over time. B) Outline of the extraction of relevant information (nuclei and aggregated cell positions) from images of histopathological brain slices. First, the grey matter is selected, then image segmentation detects aggregated cells and nuclei, and finally the cell position and cell state is reconstructed virtually. Scale bar in B: 5 mm (left), 50 µm (middle), and 5 mm (right).

Aggregated cells are capable of triggering aggregation in other cells. This could occur by transfer of aggregates that act as seeds via axonal connections, or by less direct means such as inducing inflammation,^[^ ^30^ ^]^ **Figure** **1**A. The parameters of this model include a rate constant for cell‐autonomous triggering (*k*_*a*_), a rate‐constant for cell‐to‐cell triggering (*k*_*s*_), and a characteristic length scale of cell‐to‐cell triggering (σ). As the cell‐to‐cell triggering process couples the behavior of cells across space, we also refer to this process as spatial coupling. Note that we here use the term *triggers* of aggregation to avoid connotations of a specific molecular mechanism, e.g. the spreading of aggregates from one cell to another. Our model is general, describing any process by which a sudden switch from a healthy state to a runaway aggregation state occurs, and only distinguishes a spontaneous switch, i.e. a cell‐autonomous trigger, from a switch by interactions with other cells, i.e. a cell‐to‐cell trigger.

We assume that the ability of an aggregated cell to trigger aggregation decays with distance, and here generally assume that this takes the form of a normal distribution, with a standard deviation σ, the length scale of cell‐to‐cell triggering. A normal distribution describes the situation where triggering occurs by a randomly diffusing species, but may also be used as an approximation for how the average number of axonal connections varies with distance. As we show later, the exact choice of the functional form of the distance dependence does not affect our conclusions (Figure S8, Supporting Information). This description allows us to capture the different possible mechanisms for the evolution of the spatial aggregate patterns, often referred to as *spreading*. We avoid this term here given the potential confusion as to whether it refers to the increase in the size of the region affected by pathology or the actual transfer of aggregated species. Finally, to reflect recent biological insights,^[^ ^31^, ^32^ ^]^ we investigate the effect a variable *vulnerability* term that modulates the probability of a given cell being triggered. This reflects biological variability between individual cells, such as different monomer expression levels or a varying ability to remove aggregates.

**Figure 2:**
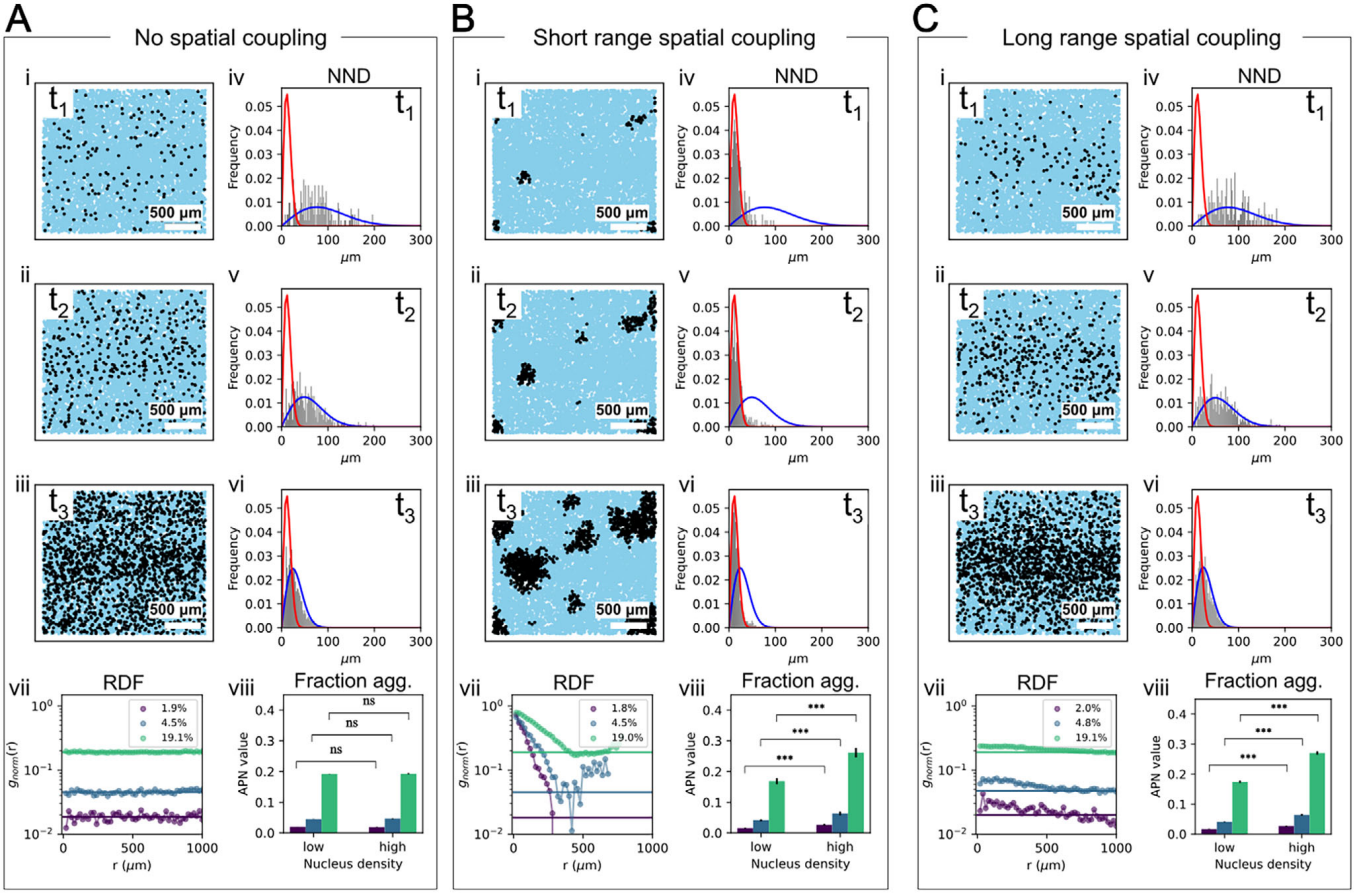
Simulation and analysis of intra‐brain region patterns under varying spatial coupling conditions and fraction of cells aggregated. A) Patterns observed with no spatial coupling, i.e. a purely cell‐autonomous mechanism, shown at three time‐points corresponding to fractions of aggregated cells of 2%, 5%, and 20%. B) Patterns with short‐range spatial coupling across the same fractions: 2%, 5%, and 20%. C) Patterns with long‐range spatial coupling across the same fractions: 2%, 5%, and 20%. In each panel, the top left sub‐panels show 2D spatial patterns at different fraction aggregated, the top right sub‐panels display the corresponding nearest neighbor distance distribution (grey histogram) with the analytical expression in the random limit (blue line) and the direct nearest neighbor coupling limit (red line), the bottom left sub‐panels present the normalized radial distribution function (points) with the fully random distribution shown as a solid line (see Method for definition), and the bottom right sub‐panels illustrate the aggregate‐per‐nucleus values in regions of varying nucleus density (n=10 simulations at each set of conditions). Statistical comparison between low and high density regions for the simulation data was performed using a two‐tailed independent samples t‐test (***p < 0.001, ns = not significant). Note the dense, compact aggregate clusters in the short‐range coupling model (B) and the more diffuse, widespread clustering seen in the long‐range coupling model (C). These visual differences are quantified by the distinct shapes of the Radial Distribution Function (RDF) curves (panel vii) and the Aggregated‐per‐nucleus (APN) values (panel viii) in each condition. Scale bar on panels i–iii: 500 µm. The simulation parameters for this figure are provided in Section Simulation Parameters.

The behavior of the model is demonstrated in **Figure** **2** for a simple cell arrangement and under a variety of conditions. We will first discuss how to quantify the distribution patterns of aggregated cells that emerge from this model and how the different processes influence them. We go on to analyse data from PSP patients to quantify rates for the different processes modeled, and identify the rate‐limiting steps. Finally, we use simulations on a virtual reconstruction of PSP brain tissue to illustrate how aggregated cell distributions evolve over the course of the disease, and to assess how varying the rates of disease processes would influence the outcome.

### 2.2 Development of Spatial Measures to Distinguish Mechanisms

Brain slices from individuals with neurodegenerative disease contain a wealth of information, not just on the quantity of neuronal tangles and other deposits of aggregated protein, but also on the spatial distribution of these structures. This spatial distribution varies in different brain regions, and at different stages of disease. From the spatial distribution of aggregated cells it is possible to infer mechanistic information on the strength of cell‐to‐cell interactions, their length dependence and the importance of de‐novo formation of aggregated structures compared to the formation triggered by nearby aggregated cells. However, extracting this information is non‐trivial given the complexity of the images, the regional variation in brain structure (differences between brain regions, white and grey matter, cortical bands etc.) and the intrinsic stochasticity of the patterns. We therefore developed means to extract the relevant information from patient data.

As a first step, the histopathological images are processed, as detailed in Section 4, to automatically determine the position of aggregated cells and nuclei, Figure 1B. These nuclei and aggregated cell positions are used in the remainder of this work; they can be directly compared to the output of our model. We now use our model to investigate different strategies to extract mechanistic information. It should be noted that the experimental data captures only a single post‐mortem time point. This means that we will only be able to determine relative rates, rather than the absolute rates. Nonetheless, this can still provide key mechanistic insight, as it is only the relative rate of processes that determines their importance in governing disease progression. If required, it is possible to estimate the absolute rate values from the average duration of disease.

To decrease the complexity of the analysis process, we now discuss the results at two length scales separately. We will first demonstrate the measures developed on simulated data and then move on to applying them to patient data.

#### 2.2.1 Nearest‐Neighbor Distributions to Investigate Interactions Between Adjacent Cells

We first focus on the fraction of aggregated cells in the immediate neighborhood of an aggregated cell, which can be assessed at the shortest length scale reliably accessible from histopathological images. We use a local measure in the form of the nearest neighbor distance (NND), which is the distance from one aggregated cell to the next nearest aggregated cell, and compare this to the NND for all cells. Determining the NND for all aggregated cells or nuclei in a brain slice yields a NND distribution.

In Figure 2 (panels i–iii in each condition) we have simulated a number of different scenarios for a simple arrangement of cells (random distribution of cells, with a denser band running through the centre inspired by the spatial variation in cell density seen in real data). In Figure 2 (panels iv–vi for each condition) we show the resulting NND distributions. These differ between scenarios, permitting mechanistic conclusions to be drawn form the NND distribution. The most pronounced differences between scenarios are expected when few cells contain aggregates. As the fraction of aggregate cells increases, the NND distributions for different mechanisms naturally converge as all cells become aggregated. We derived analytical expressions for the distribution of aggregates in the limits no spatial coupling and short range spatial coupling,^[^ ^33^ ^]^ which are shown superimposed on the example distributions in Figure 2.

Briefly, when the appearance of new aggregated cells is purely cell‐autonomous with no triggering by other aggregated cells, the NND distribution of aggregated cells is much higher than the NND of all cells (Figure 2A iv–vi). At the other extreme, when the new aggregated cells are formed predominantly by already aggregated cells triggering their direct neighbors, the NND distributions of aggregated cells closely resemble the NND distributions of all cells (Figure 2 Biv–vi). By contrast, when cell‐triggering acts over longer distances, the NND distribution of aggregated cells again resembles the case where no triggering takes place (Figure 2 Civ–vi). The NND distributions are thus a simple measure for the amount of cell‐to‐cell triggering occurring over short length scales, between directly adjacent cells. More advanced measures, such as the radial distribution function discussed below, provide additional information at longer length scales.

#### 2.2.2 Millimetre Length Scale Features can Distinguish Mechanisms

The analysis of NND distributions described above cannot distinguish the case in which cell‐to‐cell coupling occurs over a range substantially longer than the cell‐to‐cell separation (Figure 2C) from one in which formation of aggregated cells is random via a cell‐autonomous process (Figure 2A). While the short‐range distributions are comparable in both cases (Figure 2A,C panels iv–vi), at the larger length scale the difference in spatial feature size is clearly apparent (Figure 2A,C panels i–iii).

To quantify the spatial features at these longer length scales, we select two measures: the radial distribution function (RDF) and the locally averaged Aggregated cells Per Nucleus (APN) value. The APN value is defined as the local fraction of aggregated cells, i.e. the local number of aggregated cells divided by the local cell density. The radial distribution function is a commonly used measure to quantify spatial distributions. By contrast, the APN value was developed in this work to help interpret the features of the aggregated cell distribution, exploiting variation in the underlying density of cells. A plot of APN vs cell density encodes mechanistic information, on how different cell densities affect aggregate formation. As cell densities vary over a mm length scale in brains, for example, due to the presence of cortical layers, this can be exploited to gain mechanistic insights.

The APN value represents a fraction of aggregated cells, rather than an absolute number. This means it does filters out spatial features due to variations in cell density in a system where the rate of aggregate formation is unaffected by cell density. This is the case for example, when only cell‐autonomous processes take place. However, when cell density affects aggregation, for example, when cell‐to‐cell triggering depends on the separation of cells, we expect that these features will be captured in an APN vs nucleus density plot. Indeed, this is confirmed by our simulations: when cell coupling is important, denser regions show a higher fraction of aggregated cells due to the stronger coupling between the more closely packed cells (Figure 2B,C, panels viii); whereas if there is no cell coupling, the APN values are the same in regions of different density (Figure 2A viii).

The RDF measures relative density along the radial axis. In its usual physical application, the RDF is normalized by the overall density such that an RDF value of 1 at a particular distance reflects a random arrangement. In our case we instead normalise the RDF to the overall density of cells, rather than the overall density of aggregated cells. This means the value of the RDF now denotes the fraction of cells aggregated at a particular distance from another aggregated cell. We also include the RDF expected in a random arrangement, the horizontal line, to highlight the degree of clustering compared to a random arrangement. In the no spatial coupling case we find, as expected, that the measured RDF is constant and overlaps with the RDF for a random arrangement (Figure 2A vii). By contrast, in the short range coupling case, we find that the RDF peaks at short distances, with values close to 1 denoting fully aggregated regions (Figure 2B vii). The size of the clusters of densely aggregated cells is also reflected in the extent of the peaks to hundreds of µm in Figure 2B vii. In the long‐range spatial coupling case, the RDF still clearly peaks above the RDF of a random arrangement. The effect is much less pronounced than in the short‐range coupling case; as even the centre of clusters are not fully aggregated, and the clusters are significantly more spread out. The different conditions show clearly distinct patterns in the RDF, allowing us to infer mechanisms.

#### 2.2.3 Cell Types and Vulnerability

In the simplest model, all cells are assigned the same vulnerability, i.e. the same resistance against being triggered to aggregate. This is the assumption used throughout the majority of this work. However, to account for the fact that a number of different cell types are involved in the accumulation of aggregates and that there is increasing evidence for further heterogeneity within a given cell type,^[^ ^34^ ^]^ we also explored the effect of varying the vulnerability of individual cells (Figure S9, Supporting Information). We tested two types of vulnerability distributions: 1) at the one extreme we use a Bernoulli distribution where a cell can only have one of two vulnerability values, high or low; this models for example, the presence of different cell types. 2) at the other extreme we use a uniform distribution, to model a continuum of vulnerability in the system. We here assume that vulnerability does not depend on cellular location and explore a spatially dependent vulnerability below.

The results from simulating different vulnerability distributions show no qualitative changes in the NND distribution, RDF, or APN values at given fractions of aggregated cells. This means that conclusions about the dominant mechanism remain robust even when vulnerability distributions are not modeled in detail. However, the length scale of the cell‐to‐cell coupling, &sigma;, is weakly coupled to the vulnerability. Thus the absolute values of this parameter are expected to be less accurate in the absence of an accurate measure of cell vulnerability variation.

Having established the spatial features in simulation, we next consider post‐mortem data from people with PSP.

### 2.3 Mechanistic Information From Human Tissue on Different Length Scales

Tau aggregates are identified from brain slice images by image analysis and then classified into different subtypes, tufted astrocytes (TA), which are formed in astrocytes, coiled bodies (CB), which are formed in glial cells, and neurofibrillary tangles (NFT), which are formed in neurons, by a machine learning algorithm.^[^ ^24^ ^]^ Their positions, as well as all nucleus positions, are recorded and will be used in the subsequent analysis. This process is performed on all images, across stages and brain regions. For details see Experimental Section.

#### 2.3.1 Random Distribution Dominates at Cell‐Level Length Scales

**Figure 3:**
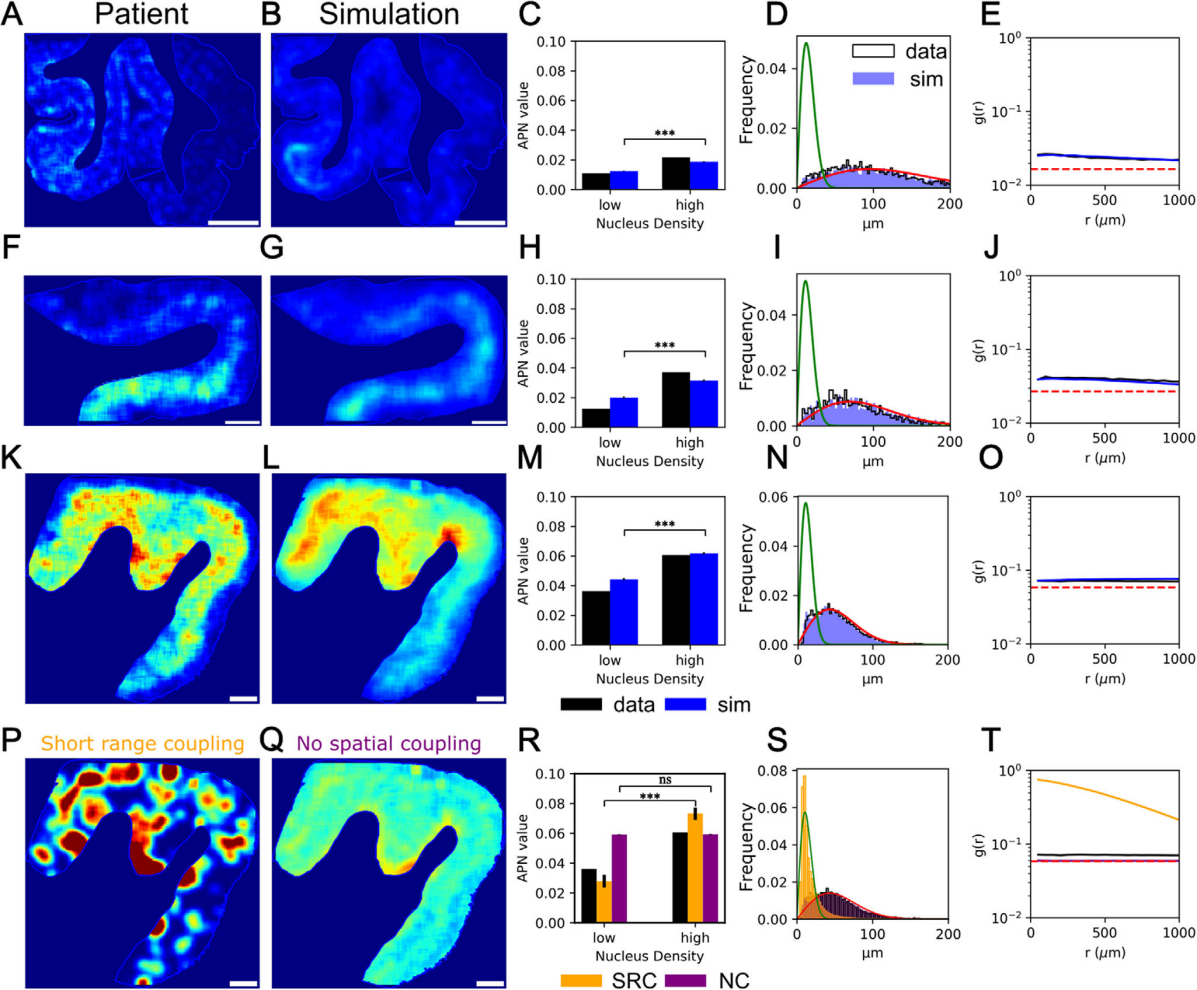
Comparison between real brain data and simulation results. Panels A–O) show comparisons between real brain data and simulations across different brain regions and the disease stages defined by Kovacs et al.^[^ ^26^ ^]^ (A), (F), and (K) display the rolling average aggregate density from real data, while (B), (G), and (L) show the corresponding simulations (average over 10 repeats) using the best‐fit parameters. (C), (H), and (M) show the aggregate‐per‐nucleus (APN) values in both low and high nucleus density regions, for the patient data (n=1 slice, black) and the simulations (blue, mean of n=10 simulations, error bar is standard error of the mean). Statistical comparison between low and high density regions for the simulation data was performed using a two‐tailed independent samples *t*‐test (***p < 0.001). (D), (I), and (N) show the nearest neighbor distance distributions for the patient data (black histogram), the simulations (blue histogram, mean of n=10 simulations), the analytical expression in the random limit (red line, see Method for the derivation) and the direct nearest neighbor coupling limit (green line, see Method for the derivation). (E), (J), and (O) show the radial distribution function for the patient data (black) and simulations (blue, mean of n=10 simulations) with the fully random RDF shown in dashed red. The patient data are from the temporal cortex at Kovacs stage 3 (A‐E), premotor cortex at stage 4 (F‐J), and primary motor cortex at stage 6 (K‐O). Panels P) and Q) show two simulation misfits to the patient data in (K), with the coupling radius set to 1/8 of the best‐fit value and the ratio *k*_*s*_/*k*_*a*_ set to 100 times the best‐fit value in (P), coloured red in R,S) and the cell‐to‐cell coupling switched off while leaving the other parameters unchanged in (Q), coloured purple in (R‐S). (R) compares APN values, (S) compares nearest neighbor distributions, and T) compares radial distribution functions between the misfits and data. Scale bar = 2 mm.

The NND distributions show that there is little coupling between directly adjacent cells and the RDFs show little variation on the length scale of ≈100 µm, regardless of brain region and stage of the disease (**Figure** **3**D, I, N, and **4**A). This finding rules out a mechanism where cells with aggregates trigger aggregation only in their closest neighbors (Figure 2B). Therefore mechanisms that are expected to transfer aggregates only to directly neighboring cells to induce aggregation there, such as tunnelling nanotubes,^[^ ^35^ ^]^ are unlikely to be dominant processes. This leaves two other possibilities: The simplest is that appearance of new aggregated cells is fully cell‐autonomous, also at longer length scales, and cells become aggregated independently of any aggregated cells in their vicinity (Figure 2A). This explanation of course would be somewhat at odds with the hypothesis of seeding, i.e. the ability of preformed aggregated tau to induce the aggregation in new cells, observed in many model systems, as well as the observation of exponential amplification of tau concentrations in disease. The other mechanistic interpretation is that inter‐cell transmission does happen, but the ability of an aggregated cell to induce aggregation in other cells is not limited to those cells close by and instead decays only slowly with distance from the aggregated cell (as in the simulated example, in Figure 2C). In this scenario aggregated cells are still responsible for triggering the aggregation, but at short length scales this *aggregation pressure* is essentially spatially uniform and the location of newly aggregated cells is governed by stochastic effects, such as the cells' differing vulnerabilities to become aggregated.

**Figure 4:**
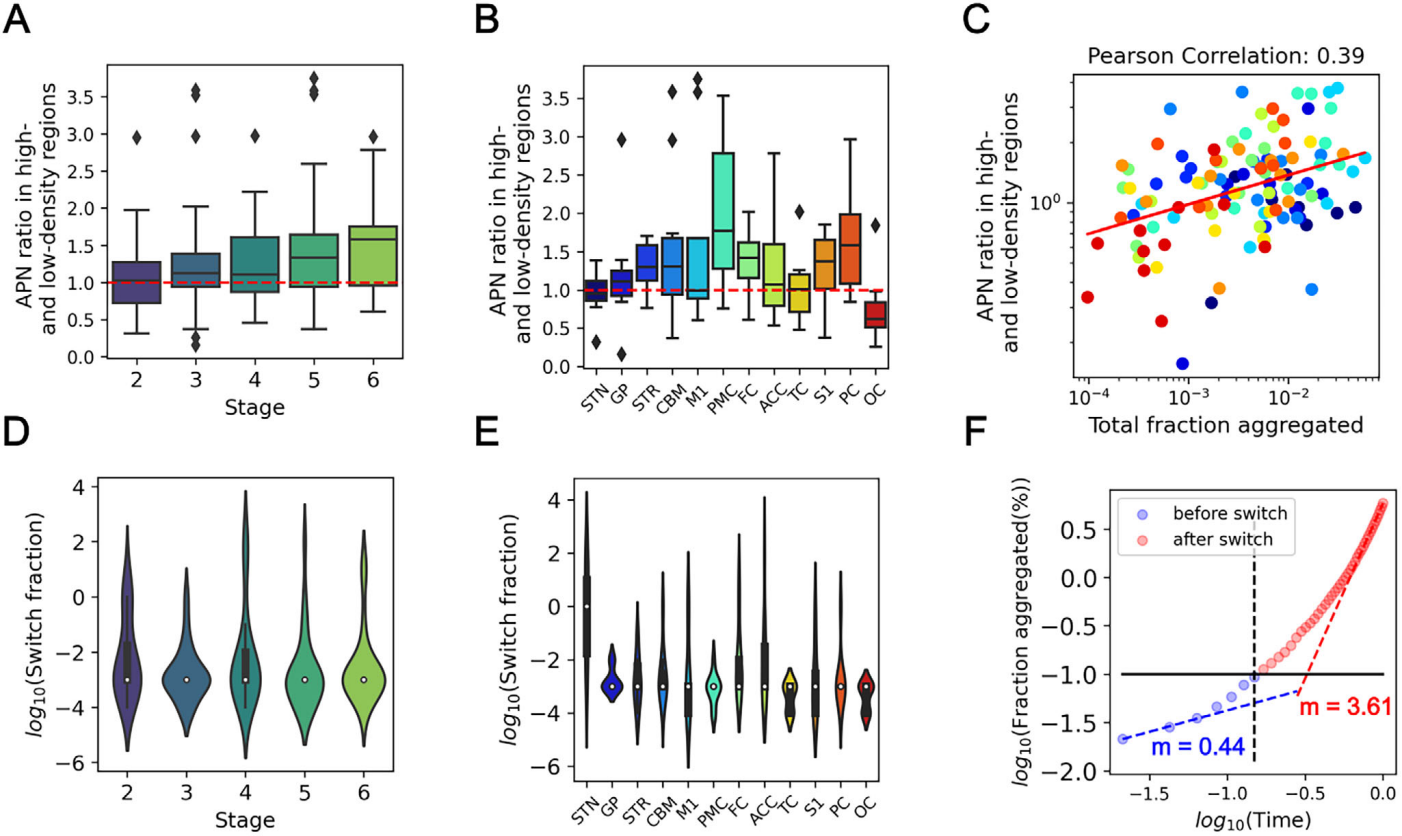
Model‐free analysis and fitted parameters from all patient data. A) Box plots summarizing the ratios in APN values across different nucleus density regions for each stage (n = 23, 22, 23, 29, and 22 samples for stages 2–6, respectively; see Table S1, Supporting Information for details). B) Box plots summarizing the ratios in aggregate‐per‐nucleus (APN) values across different nucleus density regions for each brain region (n = 10, 9, 10, 11, 9, 11, 10, 10, 8, 11, 10, and 10 for STN, GP, STR, CBM, M1, PMC, FC, ACC, TC, S1, PC, and OC, respectively; see Table S1, Supporting Information for details). For (A) and (B), the box represents the interquartile range (IQR), encompassing the middle 50% of the data with edges at the first and third quartiles. Whiskers extend to 1.5 times the IQR from the quartiles to show the data range, while points outside these whiskers are plotted as outliers. C) Correlation between the APN ratio in high‐ and low‐density regions against the fraction of aggregated cells (n = 119 total samples). The red line represents the fitted linear curve for the scattered points. Each point is colour‐coded by brain region, using the same colour scheme as shown in panel (B). D) Violin plots summarizing the switch fraction for each stage (n = 23, 22, 23, 29, and 22 samples for stages 2–6, respectively; see Table S1, Supporting Information, for details). E) Violin plots summarizing the switch fraction across different brain regions (n = 10, 9, 10, 11, 9, 11, 10, 10, 8, 11, 10, and 10 for STN, GP, STR, CBM, M1, PMC, FC, ACC, TC, S1, PC, and OC, respectively; see Table S1, Supporting Information, for details). F) Fraction of aggregated cells over time, from onset (no aggregates) up to the fraction aggregated seen in stage 6 (≈5% aggregated). Circles denote simulations based on the cell positions from the brain slice shown in Figure 3K. The solid black line is switch fraction, the dotted black line is the time when the simulated fraction aggregated reaches switch fraction. Blue and red dashed lines are the slopes of the first 3 and last 10 points, respectively, highlighting the increase in rate. Values of the slopes, *m*, are shown in the panel.

To better understand the pathogenesis at short distances we also investigated the NND of aggregated cells in different aggregated cell types separately (TAs, CBs and NFTS). The distributions resemble those observed for all aggregated cells, consistent with the previous mechanistic conclusions (Figure S5A–C, Supporting Information). Finally, we computed the nearest neighbor distance across different aggregated cell subtypes, that is the distance between for example, tufted astrocytes and the nearest coiled body (Figure S5D–I, Supporting Information). We can compute the average NND for a random arrangement of cell types and a random appearance of aggregated cells. If there was increased triggering from one type to another, for example, if most NFTs were formed by triggering from a TA, then we would expect a lower than predicted cross‐NND in the corresponding plot. In practice, we find that the average NND between two different types is slightly larger than the prediction for a random arrangement of cell types and aggregated cells (i.e. above the line of equivalence in Figure S5, Supporting Information). This indicates that specific arrangement of cell types, not appearance of aggregated cells, dominates these patterns and there is no evidence for preferential transfer of pathology from one cell type to another.

#### 2.3.2 Intra‐Brain‐Level Aggregated Cell Distributions are not Consistent With Cell‐Autonomous Triggering but Imply Long‐Range Coupling

While nearest neighbor distributions are a good measure for the spatial distributions at short length scales, to quantify the aggregated cell distributions at the longer mm‐level length scales, more complex measures that can quantify spatial features need to be employed.

In Figure 2 above we showed that the locally averaged fraction of aggregated cells (or APN value for aggregates per nucleus) and the radial distribution function (RDF) are two measures that display different features depending on the mechanism that dominates the appearance of aggregated cells. In Figure 3A,F,K we show the APN value across a brain slice, for 3 example, datasets at different disease stages. In Figure 3C,H,M we show the corresponding average APN value in high and low density cell regions. A more detailed plot, for these and additional datasets, is shown in Figure S6 (Supporting Information). In all of these images, the APN value is clearly higher in the dense cell regions than the less dense cell regions. This observation cannot be explained with a simple cell‐autonomous model, even when allowing for cell density‐dependent vulnerability as discussed below. The increased APN value in higher cell density regions however emerges naturally from a simple cell‐to‐cell coupling model, where the coupling between two cells is stronger the closer together they are.

The behavior of the RDF is also in agreement with these conclusions based on the APN‐value. The RDF‐values are significantly above the value for a random arrangement (dashed red line in Figure 3E,J,O). This suggests that cell‐to‐cell coupling plays a significant role at longer length scales, reinforcing the idea that aggregation is not merely a local event but is influenced by spatial interactions extending beyond immediate neighbors. We now move beyond these qualitative observations to extract the parameters of our model that best describe the data by fitting.

#### 2.3.3 Estimating Parameters From the Data

Using our model, we can identify which molecular mechanism dominates in human disease, and quantify the rate constants that best match the patient data. In order to do so we use the cell positions given by the histopathological images to reconstruct a virtual brain slice in silico. We can then run simulations on this specific cellular distribution to determine which set of parameter values best describe the experimental data. This allows us to determine the relative importance of cell‐autonomous over cell‐to‐cell triggering processes, given by the ratio of the cell‐autonomous triggering rate and the rate‐constant for cell‐to‐cell triggering, *k*_*a*_/*k*_*s*_, as well as the spatial coupling radius, σ.

APN histograms, rather than the full 2D images, were used to evaluate the match of simulations to data. This is motivated in part by the intrinsic stochasticity to the aggregation process: which cell will be triggered, either by cell‐autonomous processes or cell‐to‐cell coupling, is to some degree a random process. This means that individual realisations of each simulation will yield slightly different results (Figure S7, Supporting Information). This reflects the biological situation and similar stochasticity would be observed in the experimental data if it were possible to produce an exact repeat. In practice this means that simulations are unlikely to match the data cell‐for‐cell even if the model fully captured all biological processes taking place. However, higher‐level features, such as those described by the NND distribution, the RDF or the APN value for different cell density regions, are less affected by this stochasticity and therefore better suited for inference. We use the APN histogram here, but other measures, such as the radial distribution function, can also be used. The results of using RDF for inference are consistent with those presented here and are shown in the supplementary information (Figure S13, Supporting Information). While individual 2D images are subject to stochastic variation, we found that performing parameter inference by directly comparing the 2D aggregate density maps still yields results that are consistent with our other measures, and can provide a lower bound for the coupling radius (Figure S10, Supporting Information).

As shown for the example, images in Figure 3, the best‐fit simulations match the data not only at the level of the spatial measures of APN value in high and low density regions, NND distributions and RDF, but also surprisingly well at the 2D image level, see Figure 3B,G,L compared to Figure 3A, F,K, and Figures S14– S24 (Supporting Information). Some discrepancy between the empirical data and the best‐fit simulations at the level of the 2D images in Figure 3 is due to stochasticity, as discussed above. We also recognize that our model may not capture all the intricacies of the spatial dynamics of aggregation and cell interaction. However, it can explain the major features of the data well, with the best‐fit rate constants being consistent across samples (further details see next section). To put into perspective the goodness of fit, we show two misfits in Figure 3P–T. In Figure 3P we have forced the coupling radius to be 100 µ*m* and the rate ratio of cell‐to‐cell trigger and cell‐autonomous trigger to be 100 000 (compared to a best‐fit value of 800 µm and 1000), whereas in Figure 3Q aggregation is triggered only by the cell‐autonomous process and there is no cell‐to‐cell coupling. The discrepancy between the misfits and the empirical data is clear across all measures, highlighting that neither can explain the experimentally observed data.

#### 2.3.4 Trends Across Disease Stages and Brain Regions Highlight Dominance of Cell‐To‐Cell Triggers

In Figure 4, we summarise the conclusions across stages and brain regions for our dataset, which includes 11 patients, each with up to 11 brain regions (detailed numbers see Table S1, Supporting Information). We show both the results of a direct, model‐free analysis of the data, Figure 4A–C, and of the model best‐fits, Figure 4D,E.

We find that the average NND is close to that predicted when there is no coupling between nearest neighbors, at all disease stages and in all brain regions, see Figure S25A (Supporting Information). Zooming out to longer length scales reveals evidence for spatial effects. At mm length scales, there is significant variation in cell density, so the dependence of the APN value on cell density contains mechanistic information. Figure 4A and B show the ratio of the APN value in high and low nucleus density regions, grouped either by stage (A) or by brain region (B). Values above 1 denote that cells in the high nucleus density regions are more prone to aggregate than in the low nucleus density regions. This is the case in particular at later stages and is most pronounced in stages 5 and 6. The same trend is observed for the RDF, see Figure S25B (Supporting Information). When grouped by brain region, most brain regions also display a higher aggregation propensity in denser cell regions, although errors are larger given the lower number of samples in each group. The STN is an exception, with an APN ratio = 1, which might reflect biological distinctions of interest, or result from its very small volume and specific cellular conformation. The occipital lobe is the only region with APN ratio below 1. This may reflect its status as the last region to develop significant tau‐pathology under the Kovacs staging system. Consistent with this hypothesis, we show in our more detailed analysis below that the small number of aggregated cells present in the OC is not sufficient for cell‐to‐cell coupling effects to be important.

In summary, the model‐free analysis shows that aggregated cells in the immediate vicinity of a cell play little role in determining its aggregation state, yet on 100s of µm to mm the density and fraction aggregated of the surrounding cells has a significant influence. Given the apparent importance of cell density in determining aggregation propensity, we further investigated the potential mechanistic origins of this effect. There are two basic scenarios: either cells in dense regions intrinsically more vulnerable to aggregate, or the vulnerability of dense regions is simply a result of the fact that it is easier for cells to couple when they are close together. The ratio of the APN value of low and high cell density regions at different disease stages can answer this question: if the effect of high cell density regions were to simply increase the vulnerability of cells, and there was no cell‐to‐cell coupling effect, one would expect high cell density regions to be more aggregated at *any* fraction aggregated. By contrast, if the effect was due to the easier triggering of aggregation between closely packed cells, rather than an increased vulnerability in the dense regions, we would expect the difference in APN value between high and low density cell regions to become more pronounced the more aggregated the system is. This is because at a low fraction aggregated, cell‐to‐cell coupling is of lower importance, so the differences between low and high cell density regions would not be as pronounced. By plotting the ratio of the APN value in the low and high cell density regions against the total fraction of aggregated cells, Figure 4C, we see that the experimental data fall into the latter category. There is a noticeable increase in the difference between low and high cell density regions as more aggregated cells accumulate. In fact, under low aggregate conditions, there are relatively fewer aggregated cells in high cell density regions. This implies that, if there is a vulnerability difference between high and low cell density regions, high cell density regions are less, rather than more, vulnerable.

These findings support our choice of model of a spatially uniform vulnerability and a cell‐to‐cell coupling determined by cell separation. However, to explicitly test the degree to which a spatially non‐uniform vulnerability would affect results, we performed a sensitivity analysis where we introduced a spatially dependent vulnerability bias (see Figure S27, Supporting Information) and refitted an example, dataset. For spatial variations in vulnerability by 10s of percent, the same best‐fit values are recovered, showing that the best‐fit parameters are robust to some degree of spatial variations in vulnerability. Moreover, to evaluate if a spatial variation in vulnerability would lead to improved results, we compared the fitting errors at different vulnerability biases. We obtained comparable values when the vulnerability was biased by factors between 0.8 and 1.5. We can thus conclude that if a small spatial variation in vulnerability is present, it is unlikely to change the obtained best‐fit parameters, and that the inclusion of a spatial variation of vulnerability does not improve the fit quality.

Using a model of constant, spatially uniform vulnerability to fit the data by matching APN histograms (See Experimental Section Section Parameter Inference for fitting details), as outlined in the above section, we show that indeed the observed patterns can be matched. We thus obtain best‐fit values for both the relative importance of cell‐autonomous over cell‐to‐cell triggering processes, given by the ratio *k*_*s*_/*k*_*a*_ (Figure S11, Supporting Information), as well as the spatial coupling radius σ (Figures S12 and S13, Supporting Information).

While our model fits also determine the coupling radius, i.e. the characteristic length scale over which cells can trigger aggregation on other cells, the current dataset does not provide strong constraints on its value. The coupling radius most consistent with the data is in the range of several hundred µm to 1 mm (Figures S12 and S13, Supporting Information), implying that cell‐to‐cell coupling extends well beyond nearest neighbors but still resulting in spatial heterogeneity within a brain region. Note, that this value was obtained under the assumption of constant vulnerability. As discussed in the Experimental Section and shown in Figure S9 (Supporting Information), a system with spatially random varying vulnerability and a shorter coupling radius can produce similar aggregation patterns (Figure S9B, Supporting Information). Therefore, these estimates should be interpreted as effective upper bounds, given the assumption of constant vulnerability. By contrast, the switch fraction is independent of vulnerability, so its value is equally applicable also in the case of varying, spatially random vulnerability.

We use these fitted parameters to estimate the “switch fraction” (*f*_*switch*_), a key threshold where the dominant mechanism of disease progression transitions from cell‐autonomous events to cell‐to‐cell triggering. This is the point at which the rate of new aggregate formation from cell‐autonomous processes is equal to the rate from cell‐to‐cell processes. We can estimate this threshold as the simple ratio of the two rate constants: *f*_*switch*_ ≈ *k*_*a*_/*k*_*s*_ (see Experimental Section for the full derivation). Figure 4D &E shows that the switch fraction across all stages and all brain regions is on the order of 0.001, or 0.1% of cells aggregated. Figure 4C, shows that most brain regions have already exceeded the switch fraction at the time of measurement, with the exception of the OC, which is the last brain region involved in the disease progression. STN is a notable outlier in Figure 4E, due to its early involvement in disease.^[^ ^26^ ^]^ The resulting extensive cell death in this region^[^ ^36^ ^]^ renders our models, which do not include cell death, unable to fully explain the patterns.

#### 2.3.5 Two Phases Explain Key Features of Disease Progression

Our model naturally predicts two distinct phases: a slower one in which cell‐autonomous processes dominate, and a faster one in which cell‐to‐cell triggers dominate. In Figure 4F we show the simulated accumulation of aggregated cells over time, from an aggregate free state, to one that has 5% of cells aggregated (corresponding approximately to the highest levels of aggregation observed in patient data), in a virtual reconstruction of a typical brain slice. Two phases can be identified, corresponding to the cell‐autonomous phase before the switch fraction is reached, and the cell‐to‐cell phase after the switch fraction is reached. Note the much faster rate of increase in the latter parts of the cell‐to‐cell phase. We assume constant rates and vulnerability over time for this plot, in reality ageing effects may be important in particular in determining the time of onset and even further decreasing the rate of progression in the cell‐autonomous phase.

These findings imply that it is very unlikely that a single cell‐autonomous event triggers disease progression. Instead, they predict that many cell‐autonomous aggregation events take place before cell‐to‐cell mechanisms become dominant, when on the order of 0.1% of cells are already aggregated.

The change in dominant mechanism suggests that different therapeutic interventions may be effective at specific stages of the disease. When the aggregate fraction is below the switch fraction, therapies should target cell‐autonomous mechanisms. Once the switch fraction is exceeded in a brain region, interventions should focus on cell‐to‐cell coupling mechanisms. Note however that some interventions, such as reduction of the tau concentration could affect both processes equally, and that in practice it may be hard to administer therapies early enough to affect the cell‐autonomous phase. Below, we will demonstrate that targeting the wrong mechanism results in negligible effects on aggregate accumulation.

### 2.4 Simulating Effectiveness of Therapeutic Strategies

**Figure 5:**
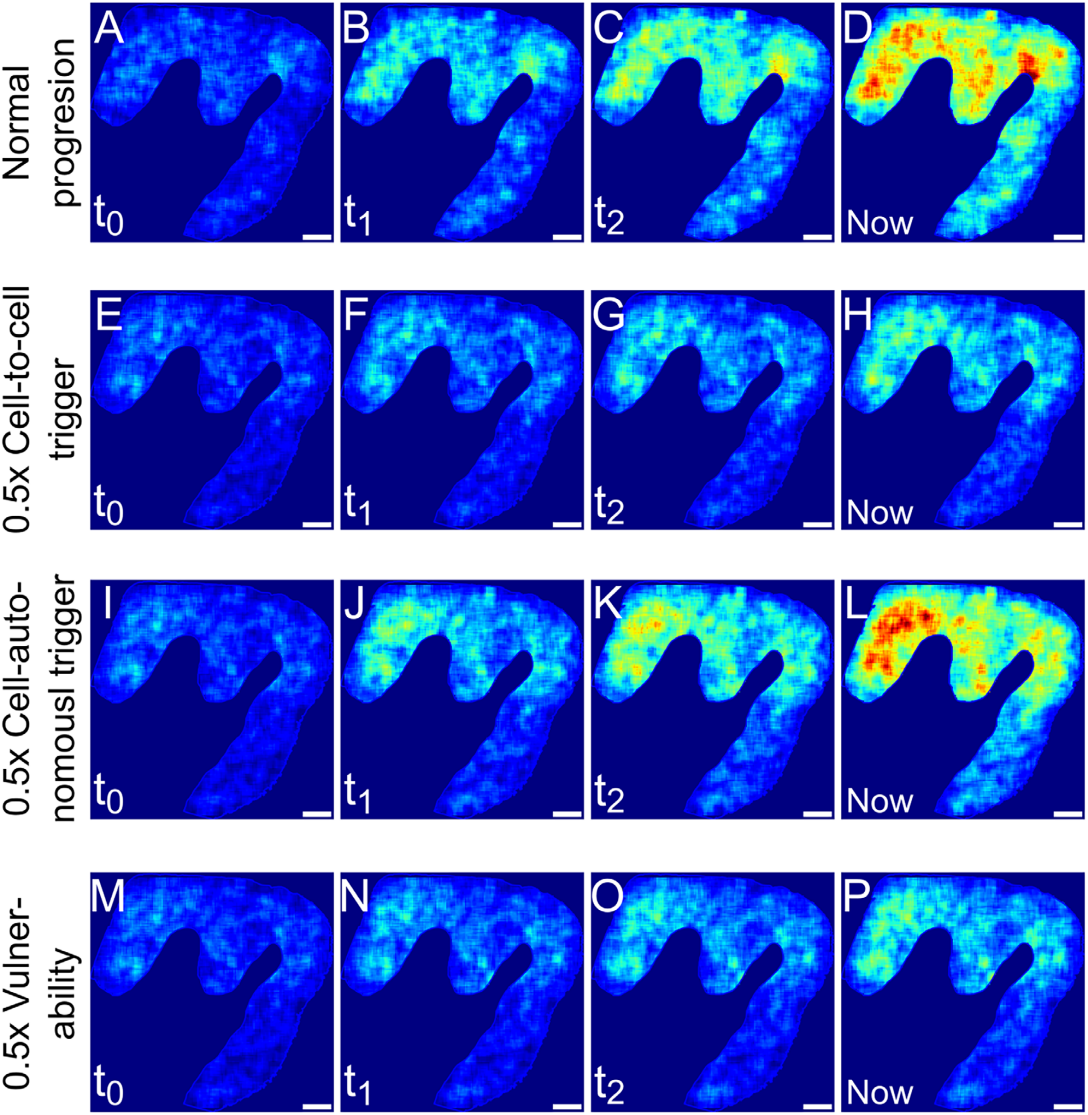
Simulations on a virtual reconstruction of a brain region. A–D) A virtual reconstruction of cell positions from measurements of brain slices allows us to simulate how the aggregate distributions may have evolved since the onset of the disease, with the last time‐point (D) matching the experimentally observed patterns. E–H) Temporal dynamics of the simulation starting from the situation in (A) with a 0.5‐fold rate of cell‐to‐cell triggering. I–L) Temporal dynamics of the simulation based starting from the situation in (A) with a 0.5‐fold rate of cell‐autonomous triggering. (M‐P) Temporal dynamics of the simulation starting from the situation in (A) with a 0.5‐fold of vulnerability. The same initial state at *t*_0_ is used in all conditions, *t*_1_ is half way between *t*_0_ and *Now*, *t*_2_ 71% of the way. Scale bar = 2 mm.

Having determined the rate constants of the individual processes in the preceding section, we can now use these insights to simulate how aggregated cell distributions may have evolved in a virtual reconstruction of each brain. Crucially we can also investigate how the distributions would be affected if a therapeutic intervention to slow a specific process were administered at different stages of the disease. We illustrate this using the primary motor cortex of a stage 6 patient in **Figure** **5**. A common initial state, *t*_0_, corresponds to 1.8% fraction aggregated or Kovacs stage 3‐4 (Figure S1A, Supporting Information), from which we start simulations under 3 conditions: with the best‐fit rate constants determined from the patient data, Figure 5A–D, with the cell‐to‐cell triggering rate lowered by 50%, Figure 5E–H, with the cell‐autonomous triggering rate lowered by 50%, Figure 5I–L, and with the vulnerability lowered by 50%, Figure 5M–P. The simulations are all run for the amount of time it takes the unaltered conditions to reach the late‐stage disease state observed in the patient data. A clear slowing in the accumulation of aggregated cells is achieved with a 50% reduction of either the cell‐to‐cell triggering rate or the vulnerability. By contrast, lowering the cell‐autonomous triggering rate by 50% leaves the progression essentially unchanged. This is evidenced by the fact that the aggregate patterns observed at the different time‐points when cell‐autonomous triggers are inhibited (row 3) are essentially the same as in the absence of inhibition (row 1). This highlights that the cell‐autonomous triggering rate becomes essentially irrelevant for the overall rate of progression, and therefore a poor drug target, once a certain fraction of aggregated cells have formed. It also showcases how modeling and simulation can be used to investigate the effect of altering different microscopic processes.

## 3 Conclusion

We have introduced a model that quantifies the mechanisms of progression of human tauopathy and tested its properties against digital pathology data. While the current work focuses on PSP as a well‐characterized single‐protein tauopathy, this framework can be easily be applied to model other neurodegenerative diseases, such as Alzheimer's disease. Moreover, inclusion of information on additional aggregating proteins, such as amyloid‐beta in Alzheimer's disease, or information on the connectivity of different brain regions are clear pathways to extend the predictive power of this approach.

Applying it to model tau aggregation in *post mortem* tissue from people with PSP, we find that, although aggregation appears random at short ranges, spatial coupling occurs over longer distances on the order of millimetres. In particular, there is an increased propensity to aggregate in dense cell regions at the later stages of PSP, consistent with the cell‐to‐cell triggering mechanism being dependent on the spatial separation of cells. The fact that these observations are explained by our minimal model suggest that the two processes we include underpin aggregate progression in PSP. Moreover, the model also predicts two phases of disease, a slower initial cell‐autonomous phase, followed by a rapid cell‐to‐cell phase. This simple mechanism can account for an increase in the rate of progression as disease advances. Finally, using the mechanisms determined from the data, we showcase in simulations how alterations of specific rates would affect the disease progression. This type of modeling can thus form the basis of prediction of drug efficacy for novel therapies to treat or prevent neurodegeneration.

## 4 Experimental Section

### Immunohistochemistry and Image Acquisition

Human post‐mortem brain tissue was acquired from the Cambridge Brain Bank. To identify hyper‐phosphorylated tau, tissue sections were immunostained using the AT8 antibody (MN1020; Thermo Scientific, USA). The pathological tau was visualized as a brown reaction product using 3,3'‐diaminobenzidine (DAB) staining. Sections were subsequently counter‐stained with haematoxylin, which visualises cell nuclei as blue reaction products. Finally, whole slide images were acquired using an Aperio AT2 scanner (Leica Biosystems, Germany) at 40x magnification.^[^ ^24^ ^]^

### Statistical Analysis

All statistical analyses were performed using Python v3.10 with the SciPy v1.14.1 library. Data are presented as mean ± standard error of the mean (SEM), unless otherwise stated in the figure legends. Box plots show the median, interquartile range (IQR), and whiskers extending to 1.5 times the IQR. The Pearson correlation coefficient was calculated to assess the linear relationship between the APN ratio and the fraction of aggregated cells (Figure 4C). A two‐tailed independent samples t‐test was used to compare the mean APN values between low and high nucleus density regions (Figure 2 and 3). A p‐value of less than 0.05 was considered statistically significant. In the figures, significance is denoted as follows: ns (not significant, p 0.05), *p < 0.05, **p < 0.01, and ***p < 0.001. The details on data pre‐processing are given in the Experimental Section and Supporting Information, a summary of sample numbers can be found in Table S1 (Supporting Information), for sample numbers used in specific plots, refer to the figure legends.

### Image Analysis Pipeline

An image analysis pipeline (Figure S4, Supporting Information) was utilized to analyse immuno‐stained brain images obtained from the Cambridge Brain Bank. The grey matter parts of the images were segmented, followed by color deconvolution to separate signals from different targets, which in this case are aggregated cells and cell nuclei. Aggregated cells and cell nuclei were then identified by thresholding the color intensity and removing artefacts. The identified aggregated cells and cell nuclei are characterized by several metrics, such as the size, the eccentricity, and the x,y position in the 2D plane. All the details of the feature extraction can be found in the work by Pansuwan et al.^[^ ^24^ ^]^ The extracted features of the objects are then further processed into nearest neighbor distance distribution (NNDD) plots and rolling density plots.

### Nearest Neighbor Analysis

To study the spatial arrangement at short distances, the nearest neighbor distance distribution (NNDD) was used. This is achieved using the cdist function from Python's scipy.spatial.distance package to calculate the distance between every possible pair of points from a lost of the positions of aggregated cells or nuclei. After calculating all the distances, we sort them for each point to identify the nearest neighbor distance for each point.

### NND Distribution for a Purely Cell‐Autonomous System

The nearest‐neighbor distance (NND) distribution describes the probability of finding the nearest aggregated cell at a distance *r* from a reference point, assumed here to be the origin. The detailed, general derivation can be found in Huang et al.^[^ ^33^ ^]^ In the case considered here the NND distribution is:

(1) $$P\left(\text{NND}\;\in \left[r,r+dr\right]\right)=2\pi rDe^{-\pi r^{2}D}dr$$

This describes the NND distribution for randomly distributed aggregated cells.

In the opposite limit, when an aggregated cell triggers its direct neighbors and there is negligible cell‐autonomous aggregation, then the brain effectively partitions into fully aggregated and non‐aggregated regions. The density of aggregated cells in the aggregated regions is simply the density of all cells, so in this limit the NND distribution is again given by the same functional form, $2\pi rD_{c}e^{-\pi r^{2}D_{c}}dr$, except that the relevant density is that of cells, *D*_*c*_ rather than the overall aggregate density as in the cell‐autonomous limit.

### Radial Distribution Function as a mm‐Length Scale Spatial Measure

The radial distribution function (RDF) describes how density varies with the radial distance *r* from a reference particle. It can be defined as

(2) $$g\left(r\right)=<\frac{dn_{r}}{2\pi rdr}>$$

where *dn*_*r*_ is the number of particles within a ring of radius *r*, and width *dr*. The RDF was used to characterize the *mm*‐length scale aggregated cell and nucleus distributions. The normalized RDF is defined as $g_{norm}\left(r\right)=\frac{g_{agg}\left(r\right)}{g_{nuc}\left(r\right)}$, where *g*_*agg*_(*r*), and *g*_*nuc*_(*r*) are RDF of aggregated cells and nuclei, respectively. Unlike the more standard definitions of the RDF, this normalized RDF achieves a values of 1 at distances where the system is fully aggregated. Moreover, the normalisation by the RDF of cells also removes contributions to the aggregate spatial patterns from the non‐uniform spatial arrangement of cells. This is crucial to obtain RDFs that are interpretable between different brain slices and images which all display different geometries. To perform these calculations, the Python package rdf2d was used, which computes the RDF based on the positions of the aggregated cells or nuclei. This function takes the particle positions and a distance interval (*dr* = 50 µm) to group the distances for analysis, returning two outputs: *g*(*r*), which contains the calculated RDF values, and *r* (radii), which are the distances at which the RDF is evaluated. These results are stored in a dictionary, with both the RDF values and corresponding radii for further analysis or storage.

### Intra‐Brain Region Analysis

**Preparation of Rolling‐Density Data** Quantifying density variation on a length scale larger than the cellular level but smaller than an entire brain region requires spatial smoothing of the discrete detections of aggregated cells. To compute the local density within a brain slice, a rolling density calculation was used. The brain slices were divided into regions according to a grid and the density of the aggregated cells was calculated in each 100 µm × 100 µm region. 100 µm as the window size was selected to avoid i) a too large window size that removes relevant spatial features, and ii) a too small window size that results in stochastic variation of density. By testing various window sizes (see Figure S26, Supporting Information), it was established that a window of 100 µm achieves the best‐fitting results. Finally, to further smooth the image, the rolling density was calculated. To do so, one grid was selected and the density values of the 7 × 7 grids area that surrounds it was averaged. The nucleus and aggregated cell densities for each brain slice are all rolling‐average pre‐processed and stored for latter usage.

**Segmentation Based on Nucleus Density** A histogram of the rolling‐averaged nucleus densities is plotted and fitted with a Gaussian distribution. The densities were classified into three groups based on the Gaussian distribution: high, medium, and low densities. The boundaries of each group were chosen based on the fitted Gaussian distribution.The first boundary was set to be the µ − 2σ, where µ and σ are the mean and standard deviation of the Gaussian distribution, respectively. The data below the first boundary is ignored to exclude the background. The second and third boundaries are set to be the mean plus/minus half of the standard deviation of the Gaussian distribution. The coarse‐grained nucleus regions are then used as a mask to separate aggregated cells into different nucleus density regions. This allows us to compare aggregated cell densities in different nucleus density regions, which provides an additional measure to characterise the aggregated cell distribution.

**APN Distribution and APN as a Function of Nucleus Density** Aggregate‐per‐nucleus (APN) value can capture local aggregation percentage without being affected by variations in cell density. It is calculated by dividing the rolling average density of aggregated cells by that of total cells. One of the important characteristics of APN value is that it can distinguish cell‐autonomous and non‐cell‐autonomous mechanisms when there is variation of cell density in the system. For a cell‐autonomous system, no matter how dense the cells are, the aggregated cell number will always be proportional to the total cell number, since the cell‐autonomous mechanism, by definition, is independent of cell‐to‐cell separation. This gives rise to a homogeneous APN value even if cell density varies. By contrast, when there is spatial coupling, high‐cell‐density regions have larger APN values than low‐density regions. As the brain slice can be segmented by the cell density, the APN value can be computed for different cell densities and used as a guide to mechanisms.

### Pseudo‐Temporal Axis

To understand the temporal evolution of the disease, a temporal dimension for the neuropathological data is needed. However, due to the variability of individuals, a universal time axis across all *post‐mortem* data is impossible. Despite such limitations, the well‐defined neuropathological staging system can give us an estimation of the disease progression. It was found that the sequential distribution of pathology is associated with the clinical severity in PSP.^[^ ^26^ ^]^ In addition to this established staging, the fraction of aggregated cells was also used to put different brain regions and individuals on a common axis of pathological severity.

### Model Construction

To capture the effect of the cell distribution in each individual, a model was built for protein aggregation in a tissue. A detailed mathematical analysis of this model, as well as it's relation to continuum models and analytical solutions in specific limits can be found in Huang et al.^[^ ^33^ ^]^ Figure 1 shows the schematics of such a model. Within the cell, in vitro protein aggregation mechanisms, including primary nucleation, growth and multiplication, govern the proliferation of protein aggregates. These aggregate formation processes may compete with removal processes. When the balance shifts to net accumulation of aggregates, or if there is a significant seeding event, the cell enters a runaway aggregation state.^[^ ^5^, ^28^, ^29^ ^]^ Based on the detailed mathematical treatment in e.g. Thompson et al. and Cotton et al.^[^ ^28^, ^29^ ^]^ we here coarse‐grain this into a switch between a healthy and a runaway aggregation state. Between cells, spatial coupling factors, such as seed transfer or inflammation, allow cells in the runaway aggregation state to exert an *aggregation pressure* on other cells to also switch from the healthy to the runaway aggregation state. In this aspect, the model closely mirrors the *Susceptible‐Infected‐Recovered* (SIR) models of epidemiology,^[^ ^37^, ^38^, ^39^ ^]^ except that a *Recovered* state is not included and the probability of “infection” is dependent on the spatial separation, which is fixed. Furthermore, different vulnerabilities can be assigned to cells, reflecting the fact that different cell types are present and that each cell may have a different level of resistance against protein aggregation. The following provides detailed explanations of each considered mechanism.

**Triggering** Triggering is the switching of a cell from the healthy state to the runaway aggregation state. This switch may for example, occur when aggregate production outweighs removal,^[^ ^28^ ^]^ or in the case when removal processes are negligible when the first self‐replicating aggregate appears either by nucleation or seeding.^[^ ^5^, ^40^ ^]^ To ensure clarity for readers who may be less familiar with aggregation kinetics, it is explicitly noted that this single term, *triggering*, was used to encompass two distinct phenomena: 1) cell‐autonomous triggering, which is a spontaneous, nucleation‐like event, and 2) cell‐to‐cell triggering, which is an induced, seeding‐like event caused by influence from a nearby aggregated cell. The model is agnostic to the molecular origin of the triggers and includes them as a stochastic process.

**Spatial Coupling Factors** There is ample experimental evidence that pathology and, at least in model systems, also aggregated species can be transferred from one cell to another, for example, along axonal connections, but potentially also through extracellular space.^[^ ^41^, ^42^ ^]^ In order to model this potential of cells in the runaway aggregating state to trigger healthy cells in a general manner, an *aggregation pressure* is defined. This was used to compute the probability to trigger a healthy cell and depends on the relative positions of the cells involved. Between brain regions, information on the connectivity exists, but on the length scales studied in this work, a determination of the connectivity of each individual cell is far out of reach of current experimental techniques. Thus, an aggregation pressure that depends only on the spatial separation of two cells were defined. The overall aggregation pressure on a given healthy cell was then computed by considering the aggregation pressures of all aggregated cells in its vicinity.

For the majority of this work, an aggregation pressure that is Gaussian in the distance between the aggregated and the healthy cell, is assumed. The aggregation pressure on a cell *i* in each time step Δ*t* is then defined as $p_{s}=1-e^{-\lambda_{i}}$, where $\lambda_{i}=\frac{1}{2\pi \sigma^{2}}k_{s}\Delta t\Sigma_{j}e^{-\frac{d_{ij}^{2}}{2\sigma^{2}}}x_{j}$ is the average aggregation events per time step Δ*t*, σ is the Gaussian diffusion radius, *d*_*ij*_ is the pairwise distance between cell *i* and *j*, *x*_*j*_ is the value quantifying whether cell *j* is aggregated (*x*_*j*_ = 1) or not (*x*_*j*_ = 0), and *k*_*s*_ is the rate of cell‐to‐cell triggering. To ensure the conclusions are general, it was also shown that changing the functional form of this effect does not change the conclusions, see Section 2.1.

**Selective Vulnerability** The model also incorporates the selective vulnerability of each cell. Though the definition of selective vulnerability varies in different contexts,^[^ ^43^ ^]^ here the selective vulnerability was defined as the cell's ability to resist the switch to a runaway aggregation state, by both cell‐autonomous or external triggers (Figure 1A). Mathematically, the vulnerability constant *v*_*i*_ was defined as a value between 0 and 1 for each cell *i*, which simply multiplies the probability of triggering to produce an updated probability that takes into account the vulnerability. In future, it may be possible to estimate the values of *v*_*i*_, for example, through spatial or single cell transcriptomics coupled with a detailed understanding of which genes are govern vulnerability. As such data are however not yet available, in the model, a number of different *v*_*i*_ distributions, such as *v*_*i*_ = *const*. or *v*_*i*_ = *U*(0, 1), where *U*(0, 1) is the uniform distribution between 0 and 1, were used.

### Simulation Parameters

The simulation parameters for Figure 2, Figures S8 and S9 (Supporting Information) are as follows. A box containing 10 002 cells was simulated. The code generates a random spatial distribution of cells while controlling the average cell‐to‐cell distance, which was set to 27.3 µm, a value chosen based on measurements from the data. A higher density (or smaller average distances) was imposed in the middle third of the y‐axis range, with an average cell‐to‐cell distance 0.8 times shorter than in the other sections, to mirror variations in cell density in real brains. The x‐coordinates are randomly and uniformly distributed across the entire x‐axis range while the y‐coordinates are adjusted randomly within each density band.

Mechanistic parameters are chosen to represent different spatial coupling conditions. For no spatial coupling, *k*_*s*_/*k*_*a*_ = 0.001 is used. For short‐range and long‐range spatial coupling, *k*_*s*_/*k*_*a*_ = 10000 is applied with σ = 40 µm for short‐range coupling and 400 µm for long‐range coupling. Periodic boundary conditions are assumed for the simulation.

### Parameter Inference

The computational model not only identifies the dominant molecular mechanisms but also allows us to quantify their rate constants using simulation based inference. To do so, a virtual brain slice was reconstructed in silico based on the cell positions from a histopathological image and then simulations on this cell arrangement were compared with experimental data using various spatial measures from the data and the simulation. The unknown parameters to be determined are the rate of cell‐autonomous triggering (*k*_*a*_), rate of cell‐to‐cell triggering (*k*_*s*_) and the cell‐to‐cell coupling radius (σ).

Because the data is an endpoint measure rather than a time‐course, we can only determine the ratio of rate constants, not their absolute values. This leaves the parameters *k*_*s*_/*k*_*a*_ and σ to be fit. To perform the fits, simulations were run with a combination of parameters {*k*_*s*_/*k*_*a*_ ∈ [0.001, 0.001, 0.01, 0.1, 1, 10, 100, 1000, 10000, 100000]}⊗{σ (µ*m*) ∈ [50, 100, 200, 400, 600, 800]}. At each set of parameters 10 repeats of the simulation were performed, to account for stochasticity. To find the best‐fit of *k*_*s*_/*k*_*a*_, we compare the histogram of APN values (all the histograms discussed are normalized to 1) from the data and the simulation. The error of each repeat of the parameter set was defined as the mean of the bin‐by‐bin squared difference between the APN histogram of this repeat and the data. The mean error was then computed across 10 repeats. The 2D plots of the mean error across different {*k*_*s*_/*k*_*a*_}⊗{σ} are shown in Figure S11 (Supporting Information). Additionally, the NND distribution was used to establish a lower bound for the spatial coupling radius σ (Figure S12, Supporting Information). The error for the NND distribution is defined as the mean of the bin‐by‐bin squared differences between the NND distribution from a single simulation and the data. Similarly, the error for the RDF is the mean of the bin‐by‐bin squared differences between the simulated and empirical RDFs and the error of the 2D image inference is the mean of the pixel‐by‐pixel squared differences between the simulated APN values and APN values from the data.

Although other readouts can be compared, such as RDF and 2D image inference (see Figure S10 and S13, Supporting Information), APN histograms and nearest neighbor distributions were selected as the readouts for fitting because they provide the best mechanistic constraints.

Given the lack of independent data on vulnerability to aggregation, a constant vulnerability was assumed in this analysis. However, the vulnerability and cell‐to‐cell coupling radius are coupled, leading to some uncertainty in the coupling radius due to the uncertainty in vulnerability. More specifically, a system with strongly varying vulnerability and a short coupling radius and a system with a weakly varying vulnerability and a large coupling radius give rise to similar patterns of aggregation, see Figure S9 (Supporting Information). The assumption of a constant vulnerability for all cells thus produces an upper bound estimate for the coupling distance.

### Derivation of the Switch Fraction

Let *f*(*t*) be the fraction of aggregated cells. The rate at which *f*(*t*) is increased due to cell autonomous processes is simply proportional to the fraction of unaggregated cells, thus

(3) $${\frac{df(t)}{dt}}_{\mathrm{cell}-\mathrm{aut}}=k_{a}\left(1-f\left(t\right)\right)$$

The contribution from cell‐to‐cell triggering is significantly more complex as it depends on the specific patterns of aggregated cells. However, for a spatially uniform system it can be assumed to be proportional to both the fraction of aggregated cells and the fraction of unaggregated cells, giving

(4) $${\frac{df(t)}{dt}}_{\mathrm{cell}-\mathrm{to}-\mathrm{cell}}=k_{s}f\left(t\right)\left(1-f\left(t\right)\right)$$

Note the similarity here to the auto‐catalytic amplification term in a Fisher‐KPP equation, as used in ref. [12] A detailed exploration of this similarity can be found in ref. [33]. The two rates are equal at the switch fraction *f*(*t*) = *f*_*s*_ thus *k*_*s*_ *f*_*s*_ = *k*_*a*_ giving an estimate for the switch fraction simply as the ratio of the rate constants as quoted in the main text.

### Ethical Approval and Patient Consent

All postmortem data analysis was conducted in accordance with the Neuropathology Research in Dementia (NeRD) ethics, with approval from the Wales Research Ethics Committee 6. All participants, or a Person in a Qualifying Relationship, provided written informed consent.

## Conflict of Interest

Georg Meisl is a consultant for WaveBreak Therapeutics.

## Supporting information

Supporting Information

## Data Availability

The data that support the findings of this study are available from the corresponding author upon reasonable request.
