# Supplementary material for: A Mathematical Model of Cellular Aggregation Predicts Patterns of Tau Accumulation in Neurodegenerative Disease
Source: Adv Sci (Weinh). 2025 Oct 27;13(1):e11297. doi: 10.1002/advs.202511297 (PMC12767001; doi:10.1002/advs.202511297)
Supplement: Supplementary file 1 — Supporting Information [file ADVS-13-e11297-s001.pdf]

# Supplementary Material

**A mathematical model of cellular aggregation predicts patterns of tau accumulation in neurodegenerative disease.**

Shih-Huan Huang[1], Annelies Quaegebeur[2], Tanrada Pansuwan[2], Timothy Rittman[2], Ruiyan Wang[1], Tuomas PJ Knowles[1], James B Rowe[2], David Klenerman[1,3,*], and Georg Meisl[1,**]

[1]Yusuf Hamied Department of Chemistry, University of Cambridge, UK
[2]Department of Clinical Neurosciences and Cambridge University Hospitals NHS Trust, University of Cambridge, UK
[3]UK Dementia Research Institute, University of Cambridge, UK
[*]Correspondence: dk10012@cam.ac.uk
[**]Correspondence: gm373@cam.ac.uk

## Inter-brain-level analysis of PSP brains

The APN value per brain region is calculated for each patient, categorised by Kovacs stages[26]. The relative aggregate amounts in different regions are approximately maintained across stages (Figure S1A). A logarithmic plot of the APN value (Figure S1 B-M) reveals consistent increase across brain regions. However, the real temporal interval between stages has not yet been determined. It is known that gliosis may lead to the increase of cell density with the progression of neurodegenerative diseases[45]. We observed similar trends of cell density increase with disease stage in our data. The mean cell densities increase from $\sim$ 1000$/mm^2$ to $\sim$ 1400 $/mm^2$, a 1.4-fold increase, from stage 2 to stage 6 (Figure S2). This increase fold also matched the value in AD (about 1.2 fold increase)[45]. Per-brain-region analysis also shows there is a correlation between nucleus density and stage in most brain regions (PMC, S1, OC, STR, M1, ACC, PC), while other brain regions (STN, GP, CMB) show no correlation or negative correlation (Figure S2). Investigation of the effect on different cell-types shows that it is predominantly oligodendrocytes that contribute to the growth of the aggregated cell percentage, whereas neuronal and astroglial aggregates show only a mild increase with stage (Figure S3A-L). This finding suggests that it is oligodendroglial cells that shape the overall evolution of pathology in the disease, which is also consistent with the recent genome wide study[46].

| Patient ID | Sex | Stage | Brain regions |
|---|---|---|---|
| 1 | Male | 2 | FC,OC,CBM,S1,M1,PMC,PC,TC,ACC,STN,GP,STR |
| 2 | Female | 2 | FC,OC,CBM,S1,PMC,PC,TC,ACC,STN,GP,STR |
| 3 | Male | 3 | FC,OC,CBM,S1,M1,PMC,PC,STN,GP,STR |
| 4 | Female | 3 | FC,OC,CBM,S1,M1,PMC,PC,TC,ACC,STN,GP,STR |
| 5 | Male | 4 | FC,OC,CBM,S1,M1,PMC,PC,TC,ACC,STN,GP,STR |
| 6 | Male | 4 | FC,OC,CBM,S1,M1,PMC,PC,TC,ACC,STN,STR |
| 7 | Female | 5 | FC,CBM,S1,M1,PMC,PC,ACC,STN,GP,STR |
| 8 | Female | 5 | FC,OC,CBM,S1,M1,PMC,PC,TC,ACC,STN,GP,STR |
| 9 | Male | 5 | OC,CBM,S1,M1,PMC,PC,ACC |
| 10 | Male | 6 | FC,OC,CBM,S1,M1,PMC,PC,TC,ACC,STN,GP,STR |
| 11 | Female | 6 | FC,OC,CBM,S1,PMC,TC,ACC,STN,GP,STR |

Table S1: Summary of patient information.

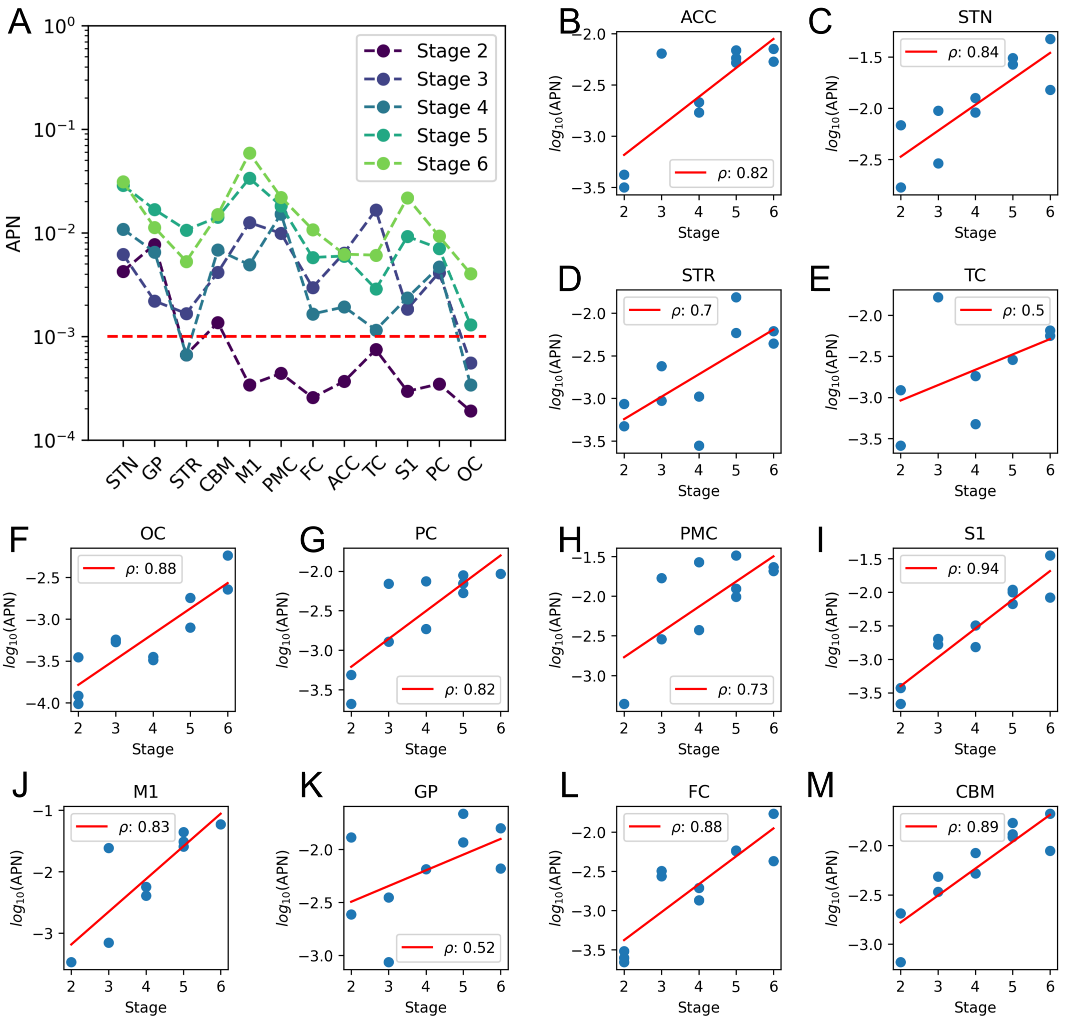


Figure S1: **Inter-brain-level analysis reveals aggregated cells increase with stage.** (A) The fraction aggregated per brain region in different disease stages. The red line is the switch fraction calculated from $k_s/k_a = 1000$ (B-M) Fraction aggregated over staged for different brain regions. $\rho$ represents the Pearson correlation coefficient.

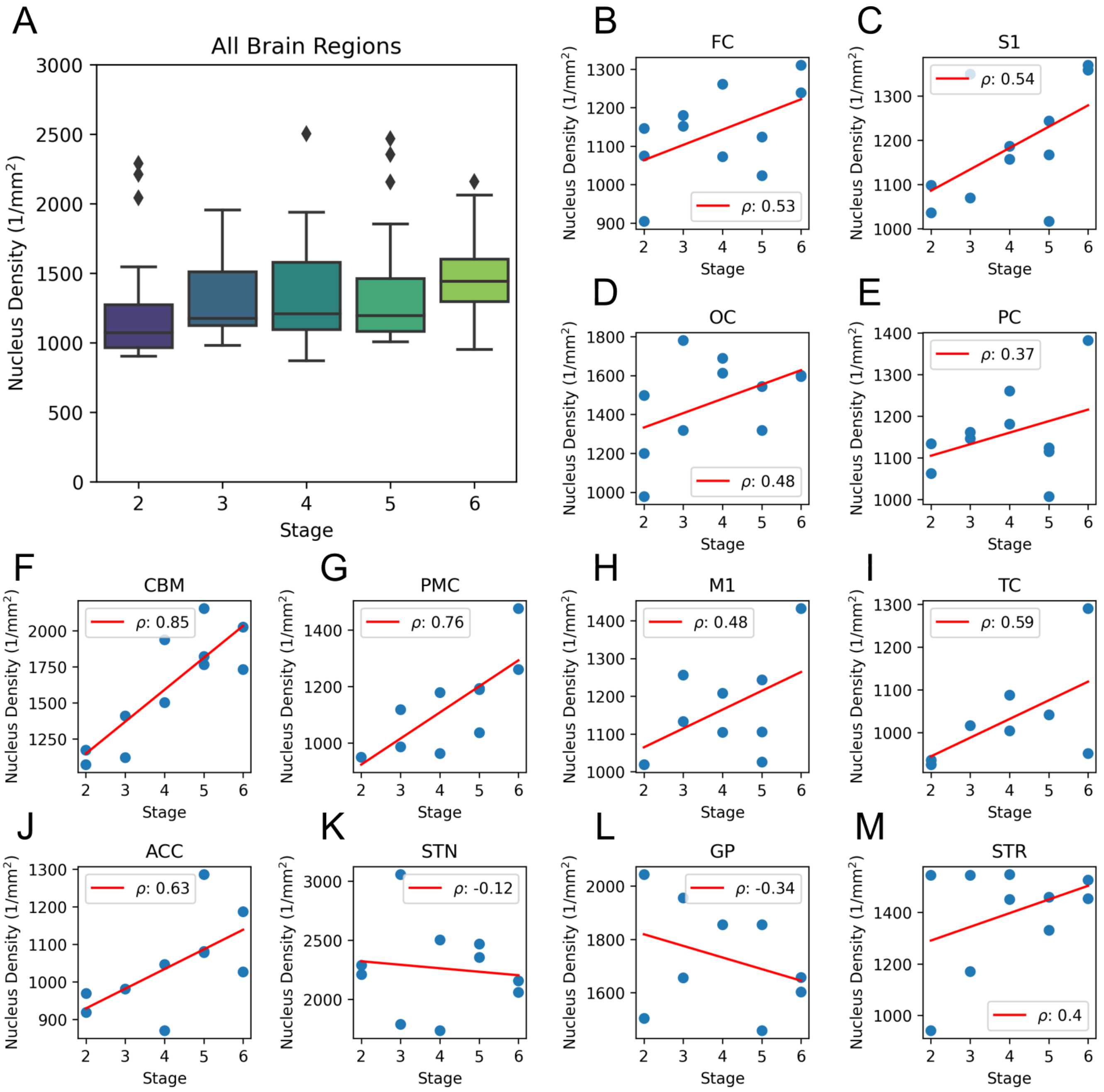


Figure S2: **Densities of cell with stage in different brain regions.** (A) The fraction aggregated per brain region in different disease stages. The box represents the interquartile range (IQR), encompassing the middle 50% of the data with edges at the first and third quartiles. Whiskers extend to 1.5 times the IQR from the quartiles to show the data range, while points outside these whiskers are plotted as outliers. (B-M) Nucleus density over stage of different brain regions. $\rho$ represents the Pearson correlation coefficient.

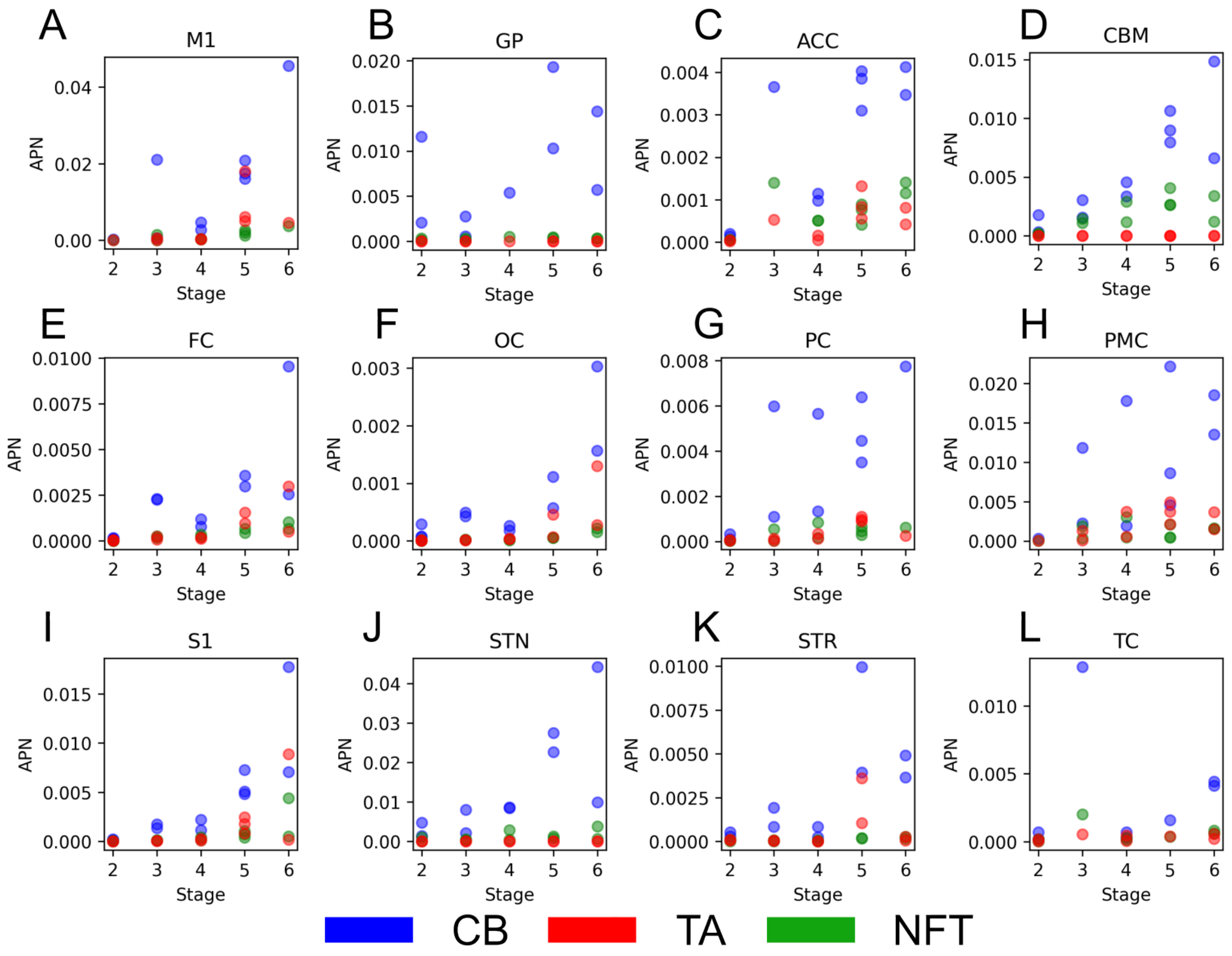


Figure S3: **APN values for different cell types across disease stages** Coiled Bodies (CBs), which form in oligodendrocytes, are shown in blue, Tufted Astrocytes (TAs), which form in astrocytes, are shown in red and Neurofibrillary Tangles (NFTs) which form in neurons are shown in green.

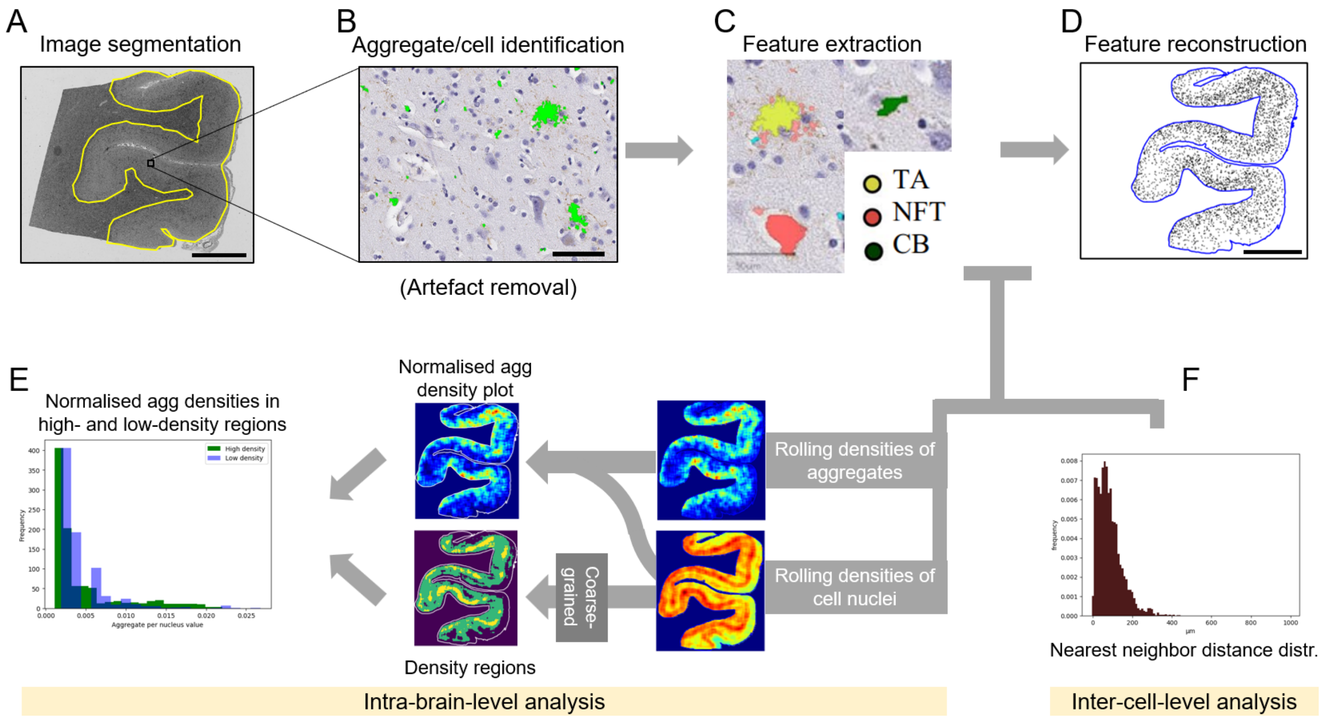


Figure S4: **Image analysis pipeline of brain slices.** (A) The region of grey matter is segmented (Scale bar = 5 mm). (B) Thresholding and shape classifiers are applied to identify aggregates and nuclei (Scale bar = 50µm). (C) Features of aggregated cells and nuclei, such as size and the spatial locations, are extracted. (D) Finally, a feature-tailored image can be reconstructed (Scale bar = 5 mm). (E & F) Aggregated cell/nucleus features can be further analysed: nearest neighbour distance distribution and rolling density plots can characterize aggregated cell/nucleus patterns on different length scales.

| | |
|---|---|
| FC | Frontal cortex |
| OC | Occipital cortex |
| CBM | Cerebellum |
| S1 | Primary somatosensory cortex |
| M1 | Primary motor cortex |
| PMC | Premotor cortex |
| PC | Parietal cortex |
| TC | Temporal cortex |
| ACC | Anterior cingulate cortex |
| STN | Substantia nigra |
| GP | Globus pallidus |
| STR | Striatum |
| PSP | Progressive supranuclear palsy |
| AD | Alzheimer's disease |
| NFT | Neurofibrillary tangle |
| CB | Coiled body |
| TA | Tufted astrocyte |

Table S2: List of abbreviations.

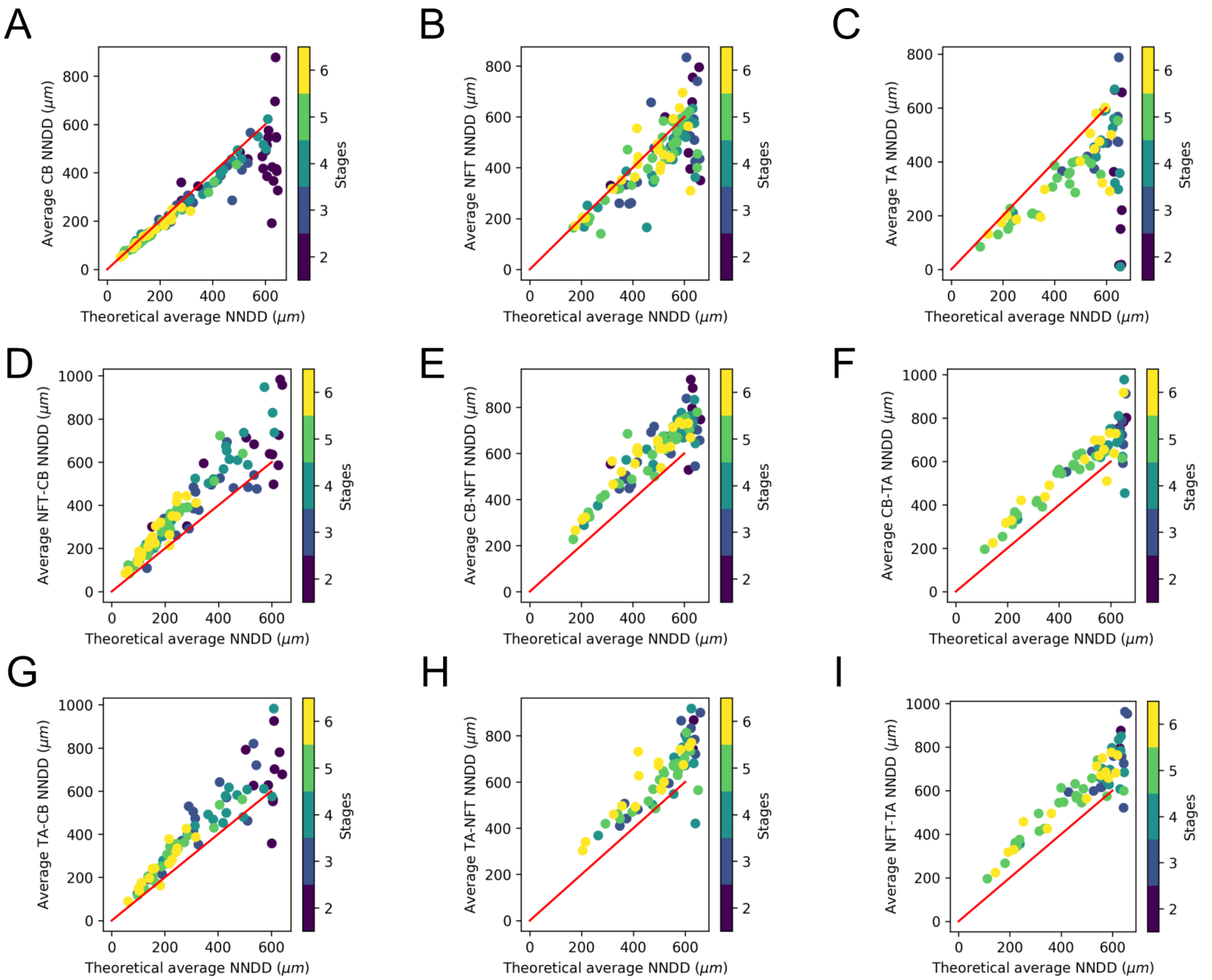


Figure S5: **NND distribution within and across different aggregated cell subtypes.** The theoretical average NNDD is plotted against the value determined for each brain slice. The theoretical average NNDD for A-C is calculated assuming a random distribution of that particular aggregated cell subtype. The theoretical average NNDD for D-I is calculated assuming a random distribution of the latter aggregated cell subtype. For example, the NFT-CB cross-type NNDD uses the theoretical random distribution of CB.

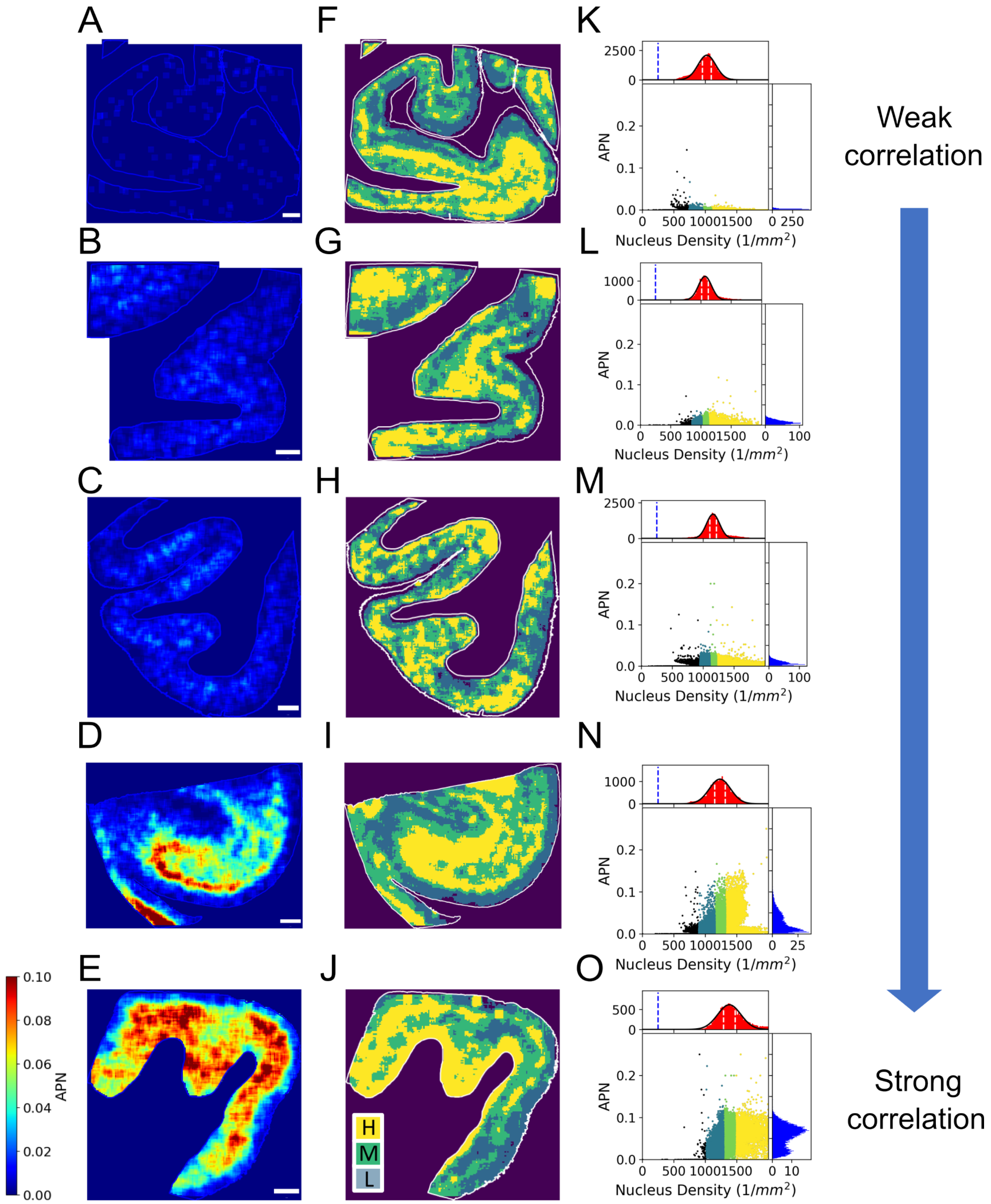


Figure S6: **Correlation between APN values and nucleus density with disease stage.** (A-E) APN plots for example brain images from Kovacs stage 2 (A), stage 3 (B), stage 4 (C), stage 5 (D) and stage 6 (E). Scale bar = 2 mm. (F-J) Corresponding nucleus density regions of (A-E). High density region: yellow; moderate density region:green; low density region: cyan. (K-O) Pixel-wise correlation plots between (A-E) and (F-J) and their corresponding histograms in two axes. Different colours show different nucleus density regions.

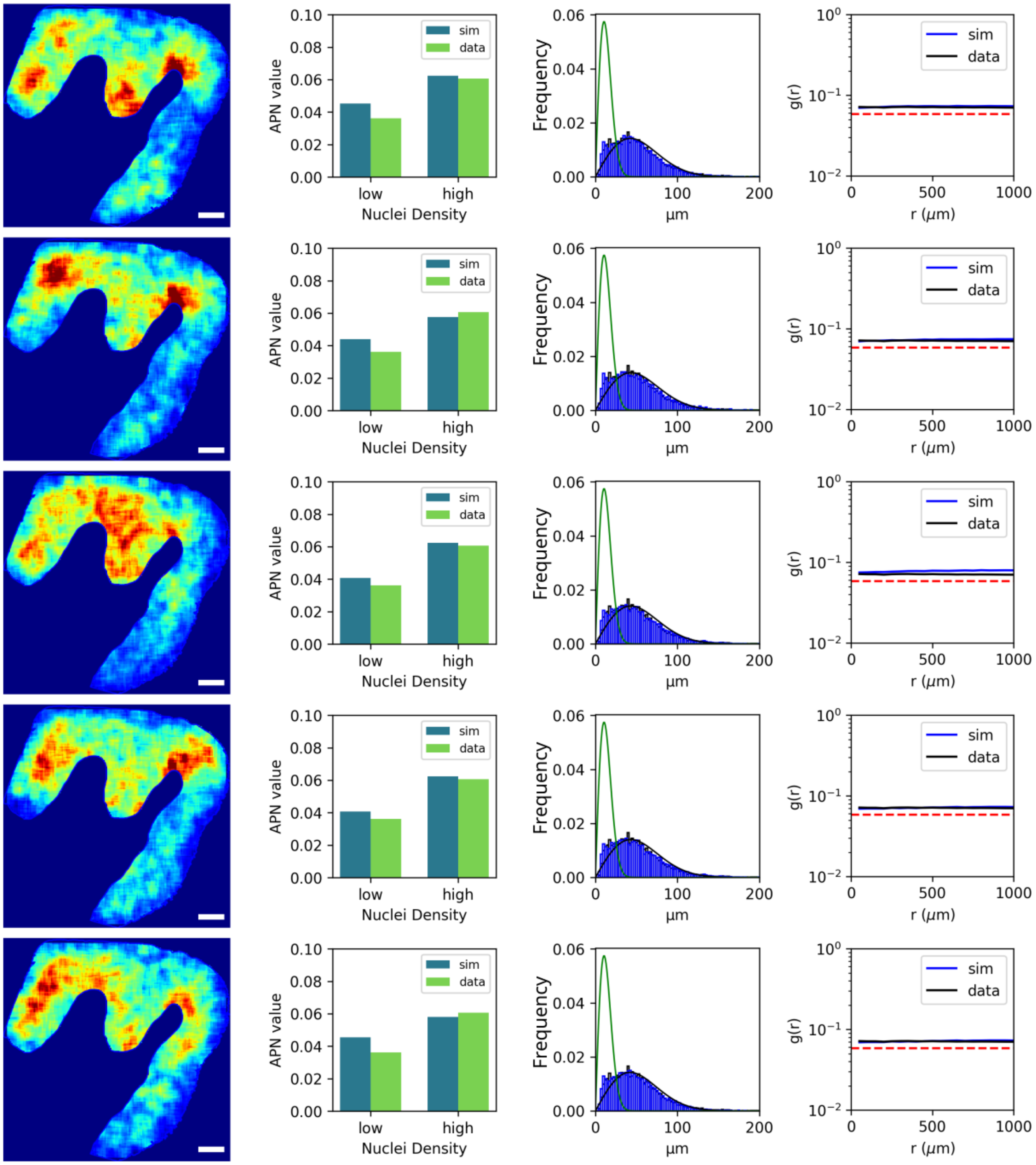

Figure S7: **Comparison of repeats of an example simulation.** Each row represents one realization of the simulation with the same parameter set. Each row contains the following: 2D patterns of the aggregated cell rolling-average density (far left), APN values in different density regions (middle left), NND distributions (middle right), and RDF (far right).

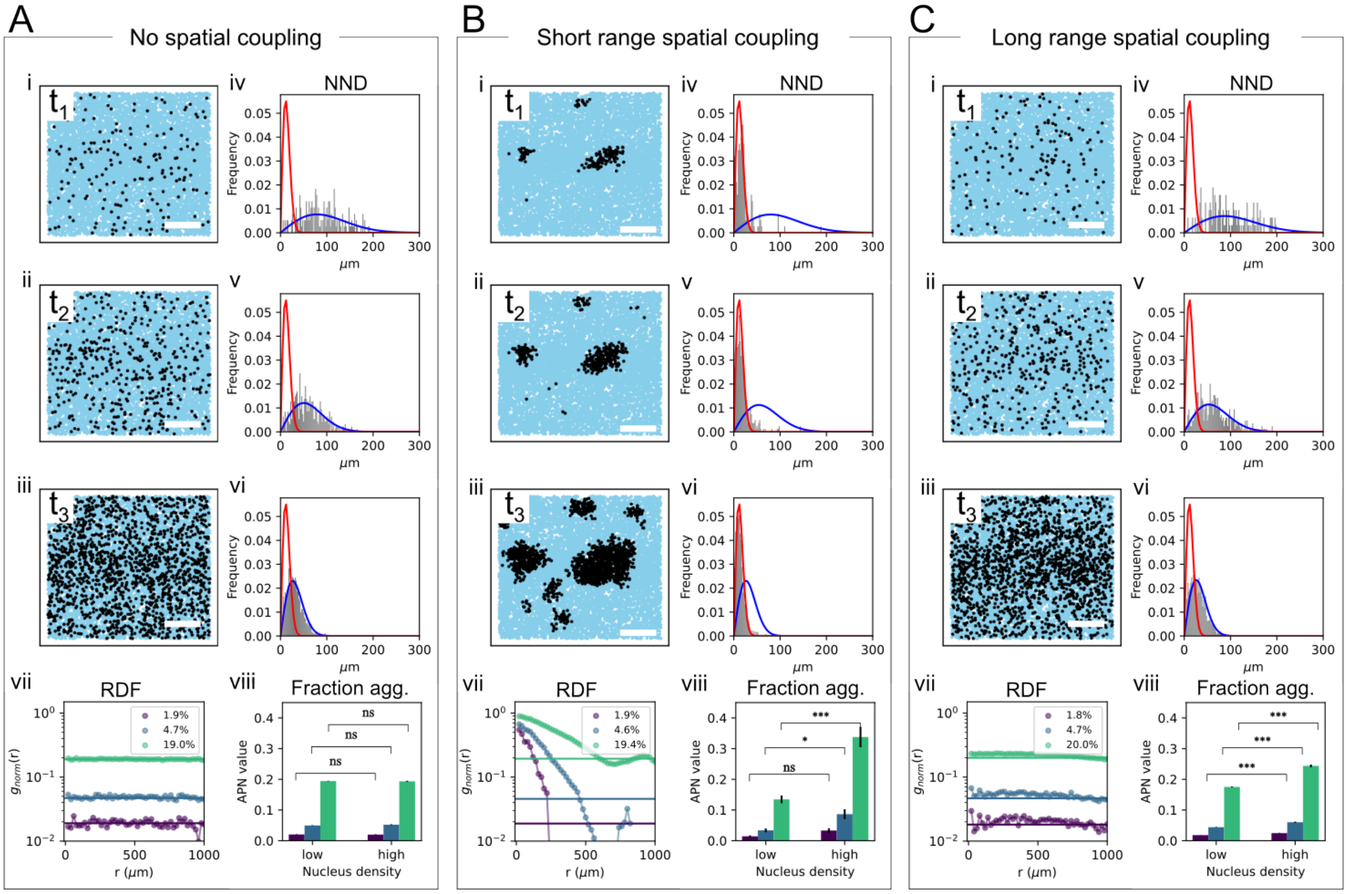


Figure S8: **Simulation of aggregation dynamics with exponential decay spatial coupling dependence** The simulation conditions are the same as in Figure 2 except that the spatial coupling has distance dependence has been changed to $e^{-d/\sigma}$ . Thus the spatial coupling strength at a distance of 0 matches between the normal distribution used in the main text, see e.g. Figure 2) and the exponential decay used here. Scale bar on panels i-iii: 500 µm. The simulation parameters for this figure are provided in Sec. *Simulation parameters*.

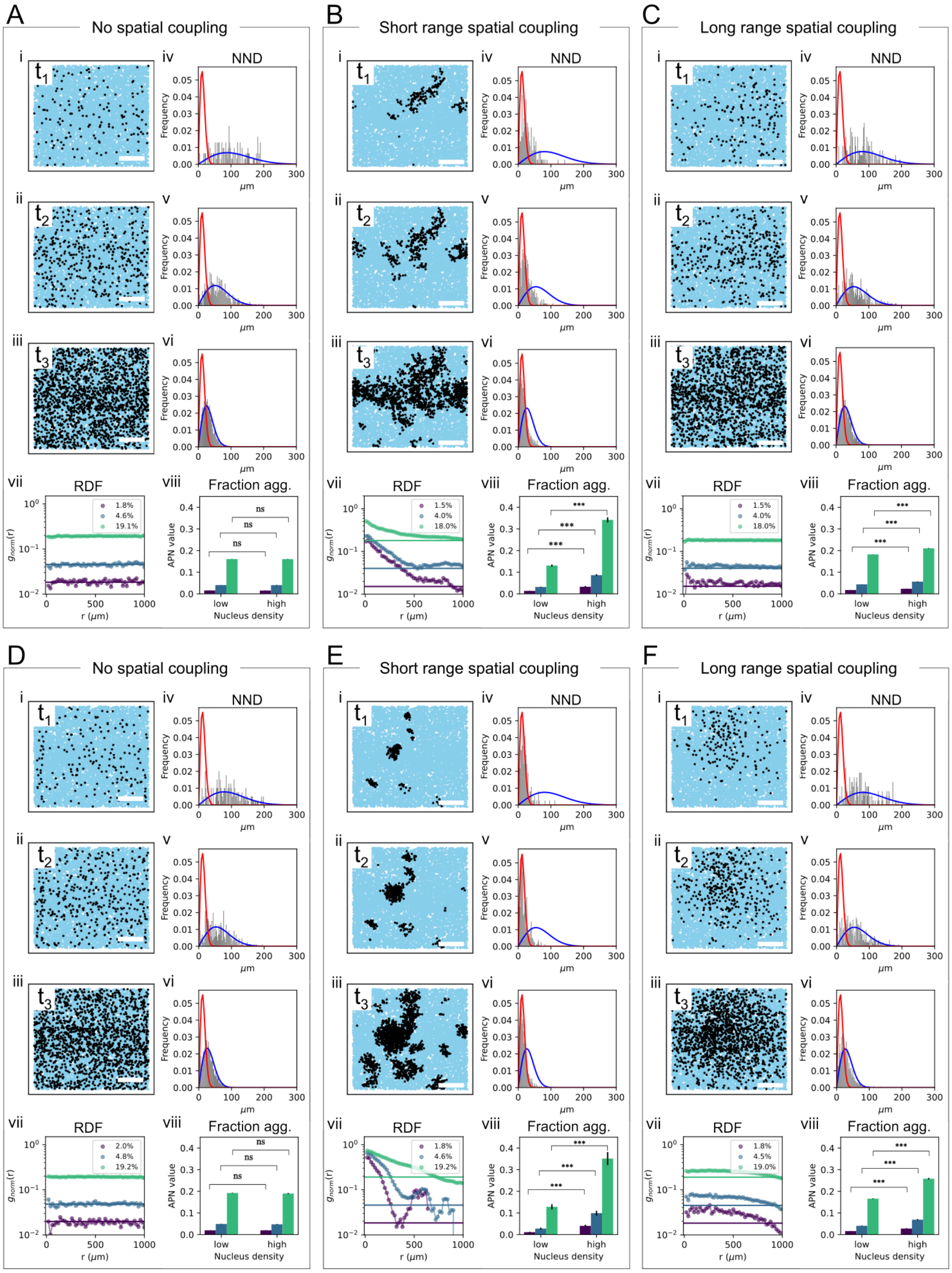


Figure S9: **Simulation of aggregation dynamics for Bernoulli and uniform distributions of vulnerability.** The simulation conditions are the same as in Figure 2 except for vulnerability values. (A-C) There are two groups of cells with distinct vulnerability values: one group with a value of 0.01, comprising 99% of the population, and another group with a value of 1, comprising 1% of the population. (D-E) the vulnerability distribution is uniform on $[0, 1]$, $U(0, 1)$. Scale bar on panels i-iii: 500 µm. The simulation parameters for this figure are provided in Sec. *Simulation parameters*.

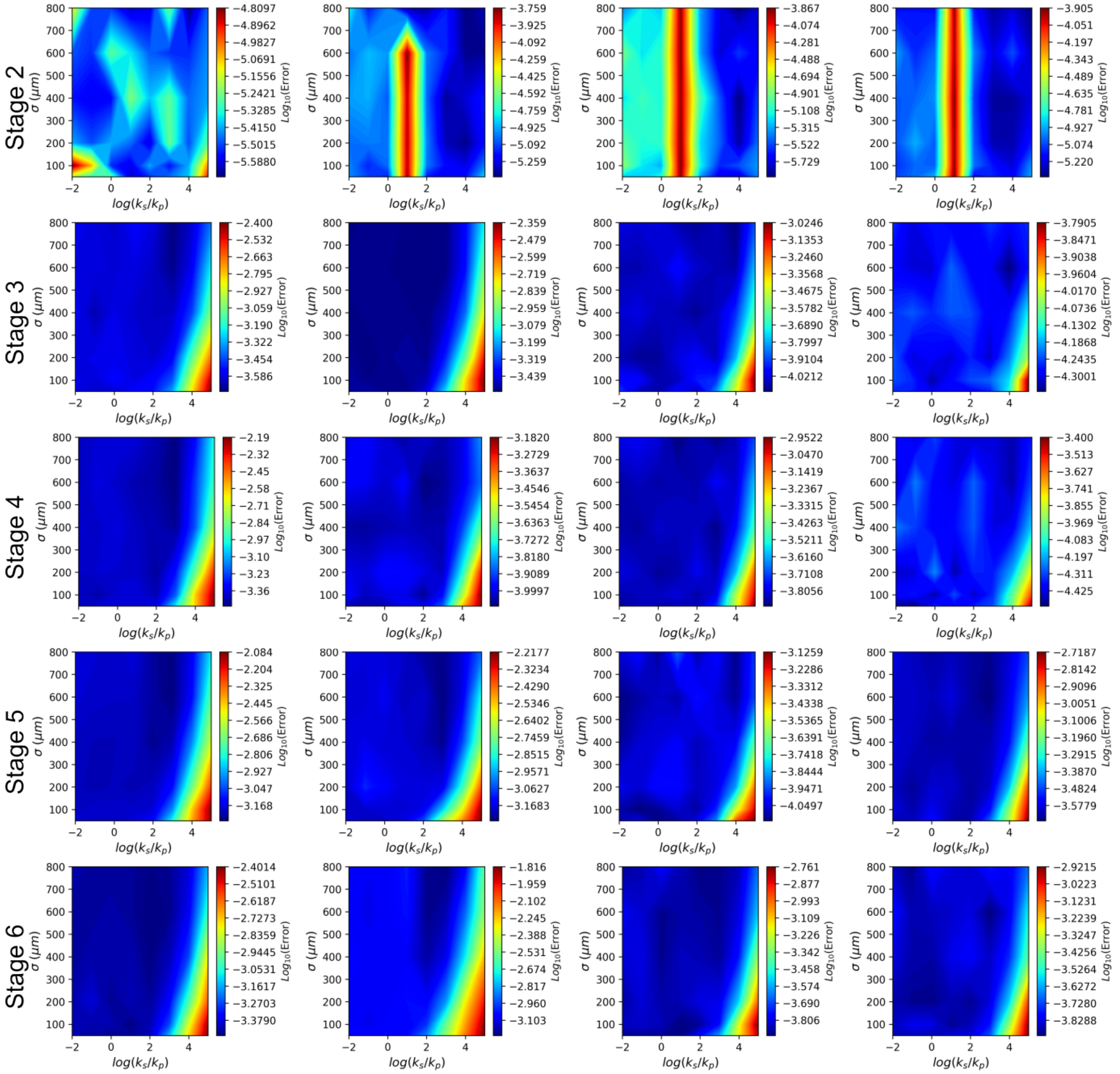


Figure S10: **Parameter inference through 2D images.** Each panel shows the two-dimensional plots of the mean error across different parameter sets (see *Parameter inference* for the definition of error). The figure shows the analysis of 20 brain slices arranged in 5 stages, each with 4 panels per stage. Each column represents a distinct brain region: from left to right PMC, M1, PC, and S1.

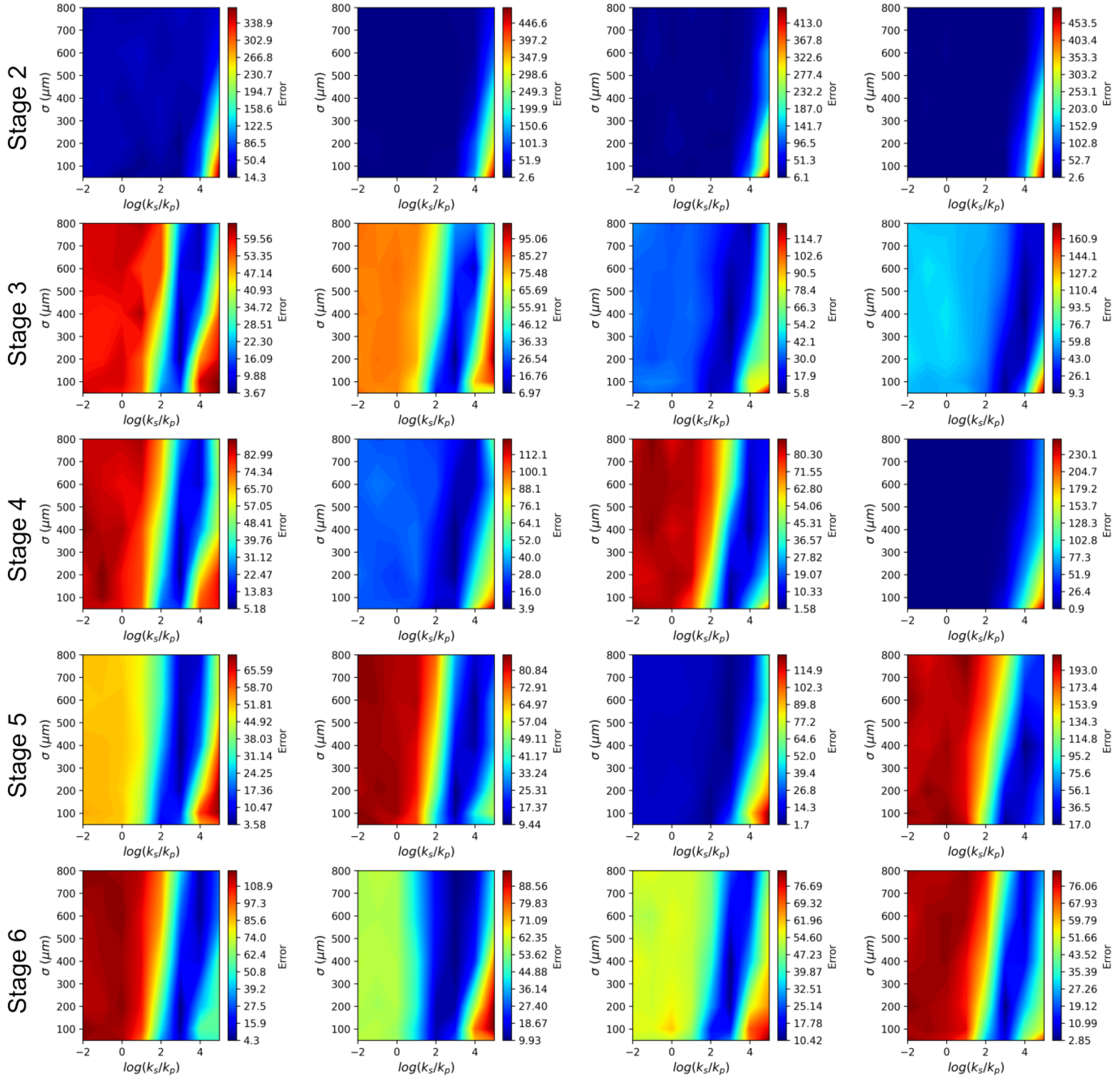


Figure S11: **Parameter inference through APN histograms.** Each panel shows the two-dimensional plots of the mean error across different parameter sets (see *Parameter inference* for the definition of error). The figure shows the analysis of 20 brain slices arranged in 5 stages, each with 4 panels per stage. Each column represents a distinct brain region: from left to right PMC, M1, PC, and S1.

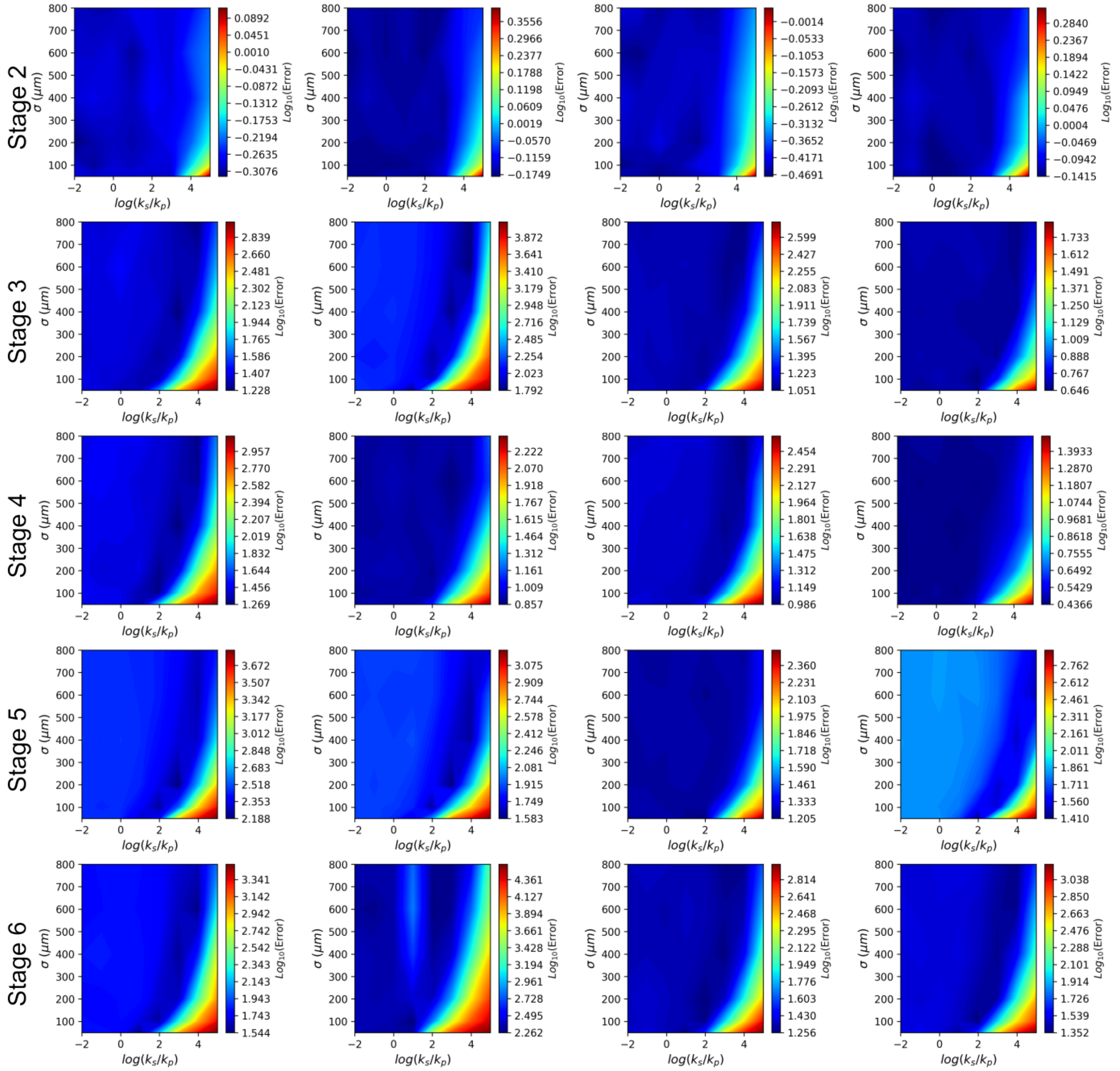


Figure S12: **Inference through NNDD.** Each panel shows the two-dimensional plots of the mean error across different parameter sets (see *Parameter inference* for the definition of error). The figure shows the analysis of 20 brain slices arranged in 5 stages, each with 4 panels per stage (corresponding to the same brain slices as in Figure S11). Each column represents a distinct brain region: from left to right PMC, M1, PC, and S1.

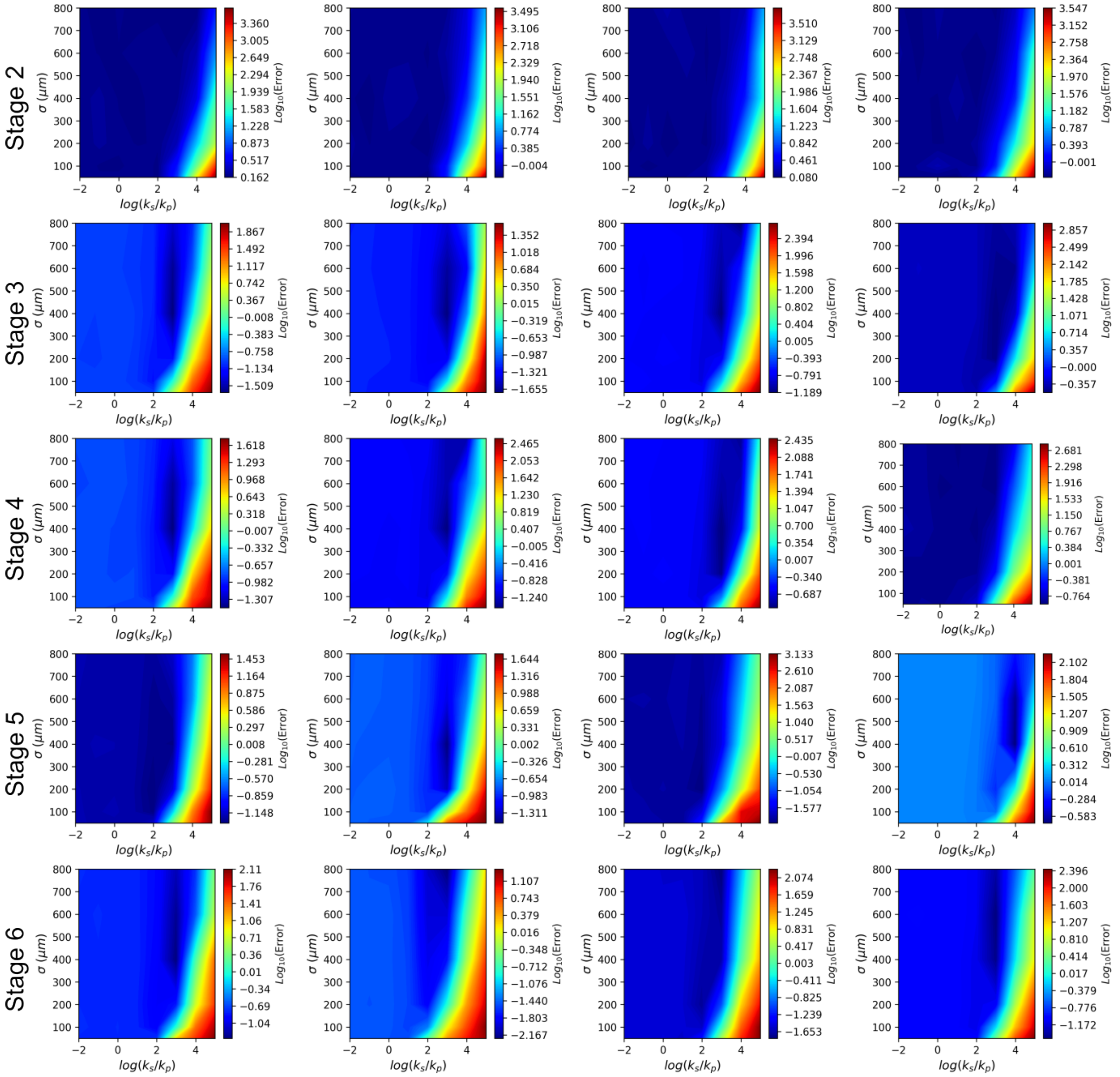


Figure S13: **Inference through RDF.** Each panel shows the two-dimensional plots of the mean error across different parameter sets (see *Parameter inference* for the definition of error). The figure shows the analysis of 20 brain slices arranged in 5 stages, each with 4 panels per stage (corresponding to the same brain slices as in Figure S11). Each column represents a distinct brain region: from left to right PMC, M1, PC, and S1.

## All data with simulations

This section shows all data as 2D heatmaps of aggregate concentrations, all on the same colour scale for comparison across the dataset. Two sets of simulations are shown, with a coupling distance of 400 µm and a coupling distance of 800 µm, and the best fit rate constants $k_s/k_a = 1000$. Some points to note: The simulations are averages over 10 repeats, to reduce stochasticity, however, this makes them appear generally smoother than the data. As detailed in the main text, the comparison of 2D heatmaps is susceptible to stochastic differences, so these comparisons should be interpreted as an approximate qualitative check. Similarly, the aggregate concentration in many samples is relatively low, limiting the utility of a visual comparison. Therefore, the quantitative fitting is performed using the spatial measures less susceptible to stochasticity and able to pick out general patterns, as detailed in the main text.

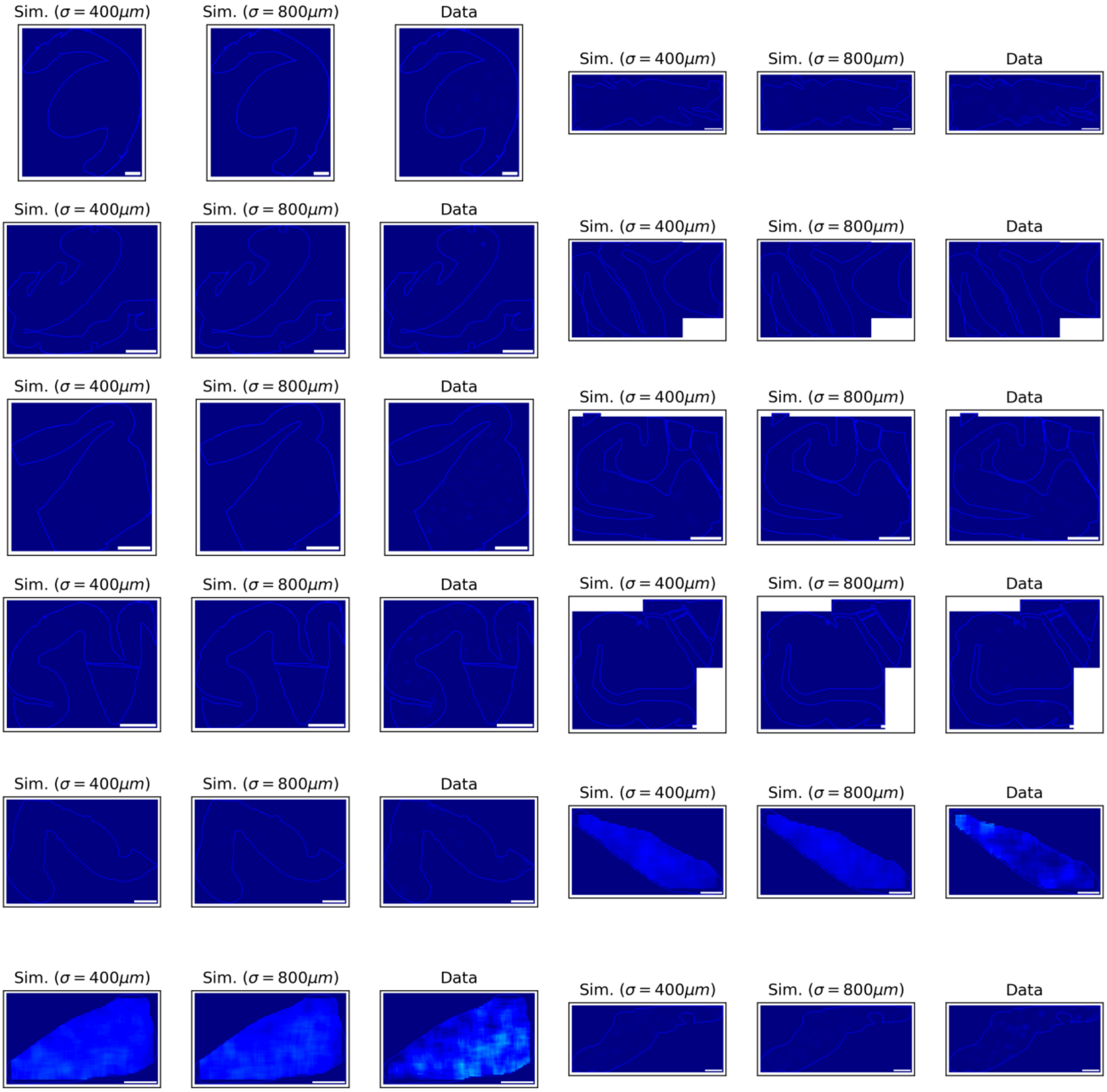


Figure S14: **Additional 2D image comparisons (Stage 2, patient ID = 1).** The simulation columns show the rolling average aggregate density from real data. The data columns show the corresponding simulations using the best-fit parameters. Scale bar = 2 mm.

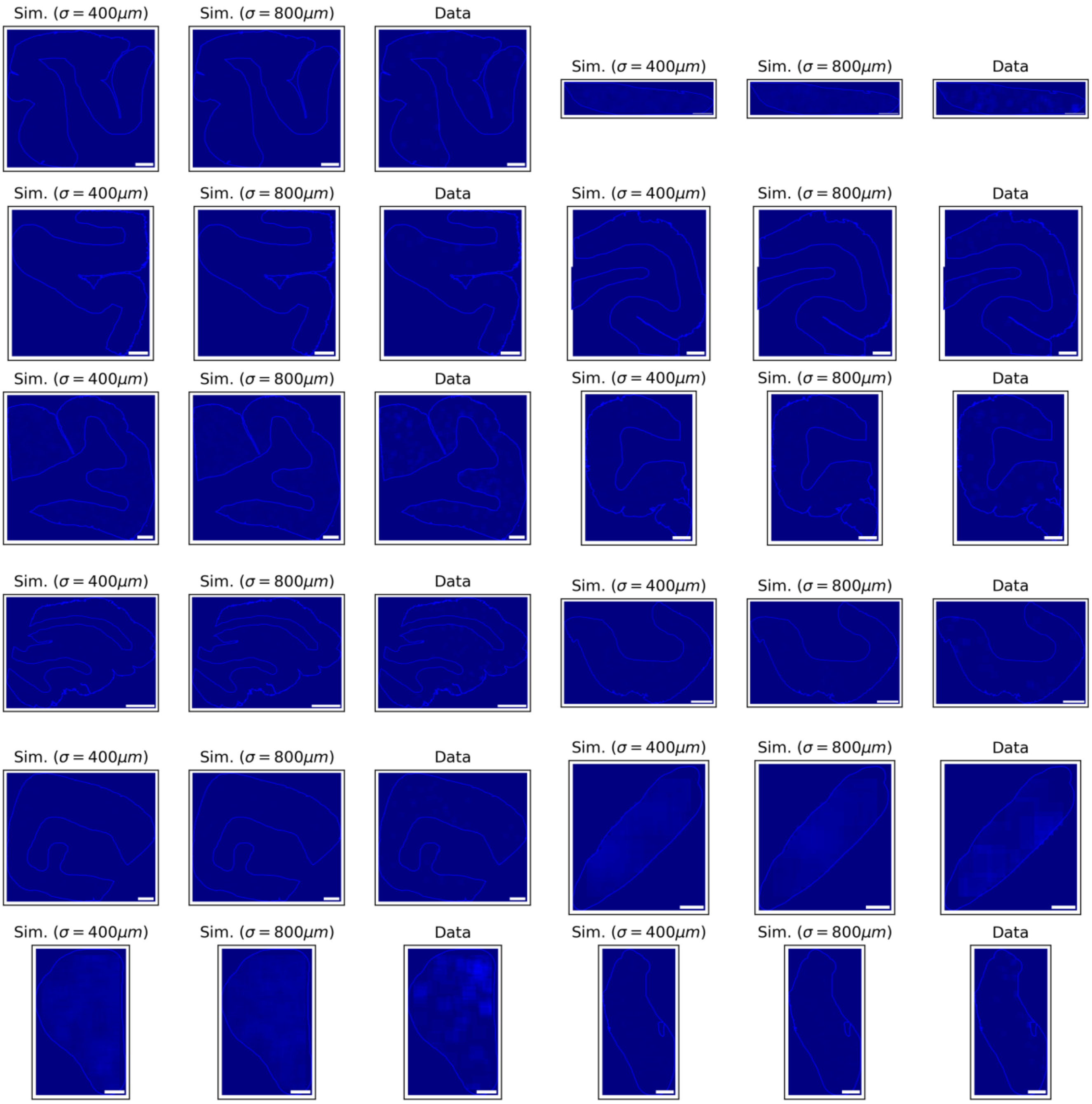


Figure S15: **Additional 2D image comparisons (Stage 2, patient ID = 2).** The simulation columns show the rolling average aggregate density from real data. The data columns show the corresponding simulations using the best-fit parameters. Scale bar = 2 mm.

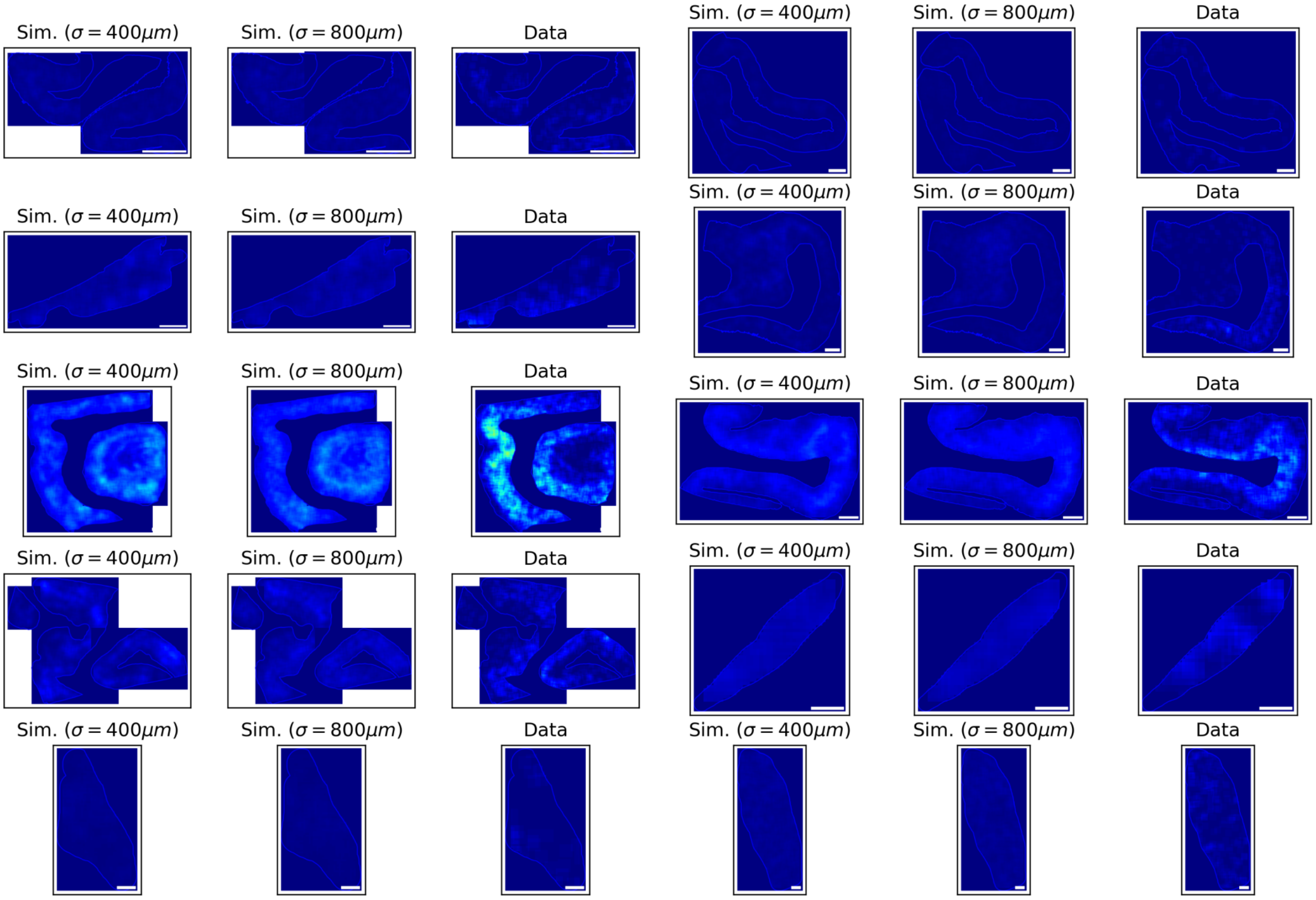


Figure S16: **Additional 2D image comparisons (Stage 3, patient ID = 3).** The simulation columns show the rolling average aggregate density from real data. The data columns show the corresponding simulations using the best-fit parameters. Scale bar = 2 mm.

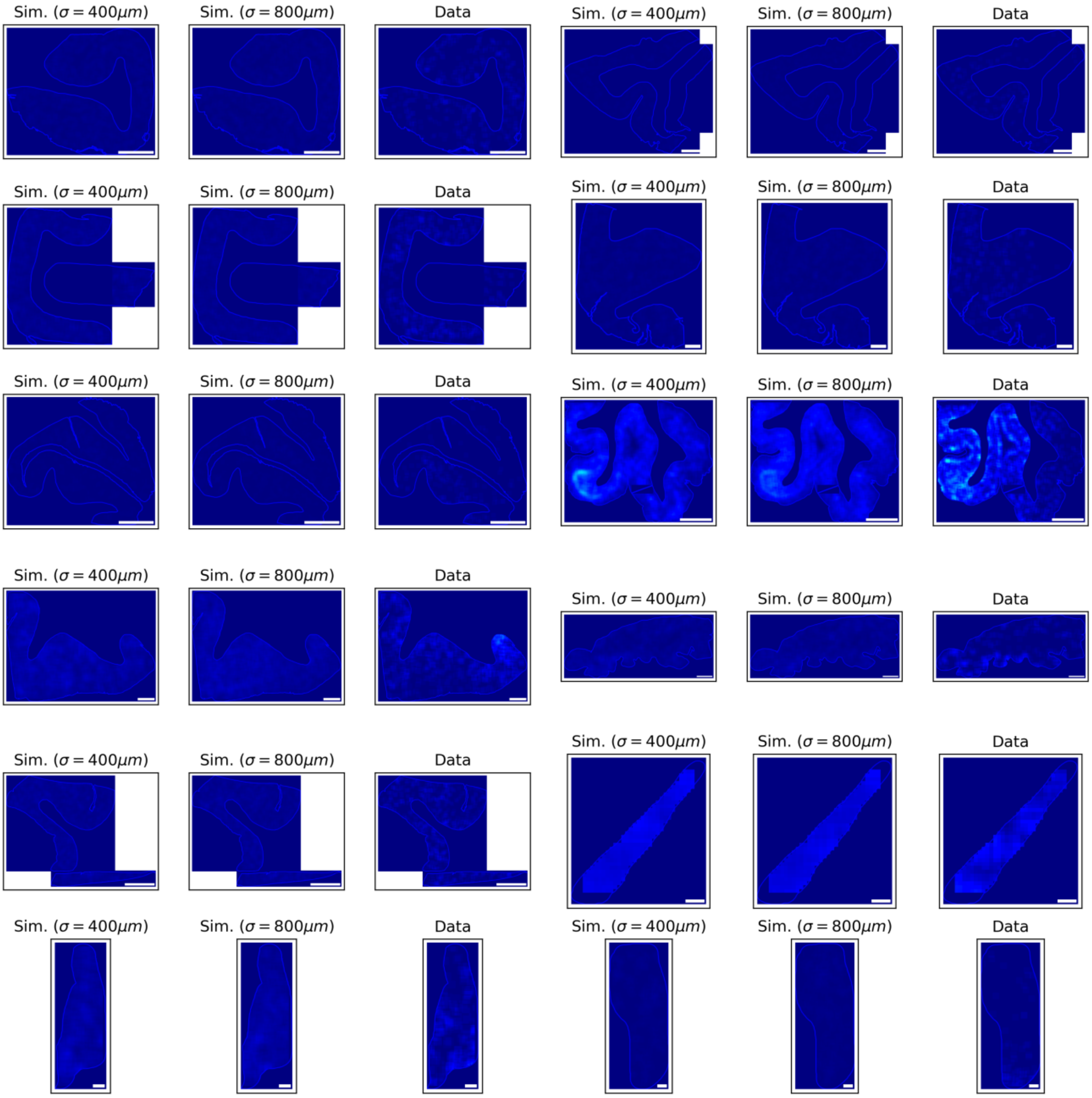


Figure S17: **Additional 2D image comparisons (Stage 3, patient ID = 4).** The simulation columns show the rolling average aggregate density from real data. The data columns show the corresponding simulations using the best-fit parameters. Scale bar = 2 mm.

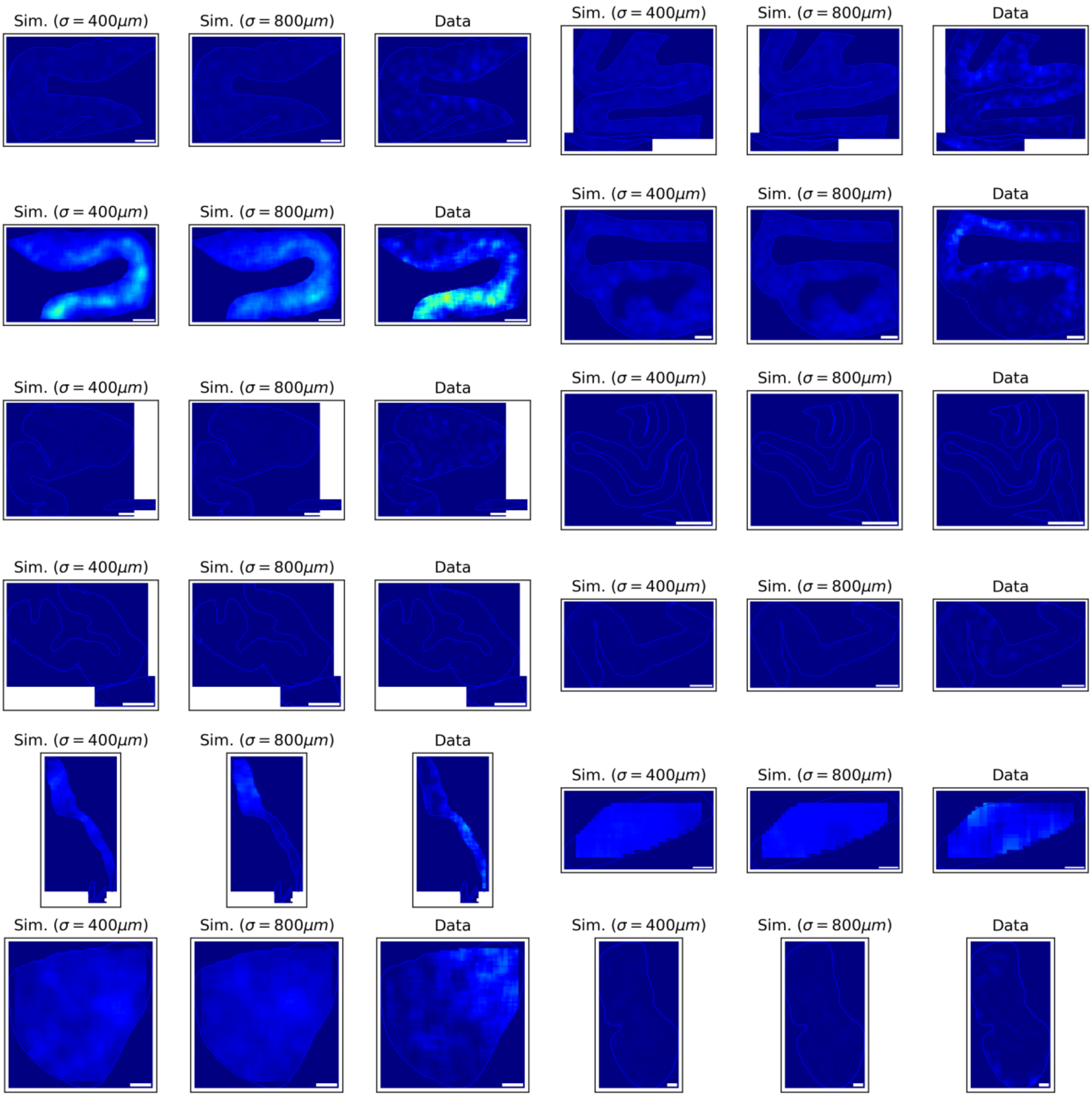


Figure S18: **Additional 2D image comparisons (Stage 4, patient ID = 5).** The simulation columns show the rolling average aggregate density from real data. The data columns show the corresponding simulations using the best-fit parameters. Scale bar = 2 mm.

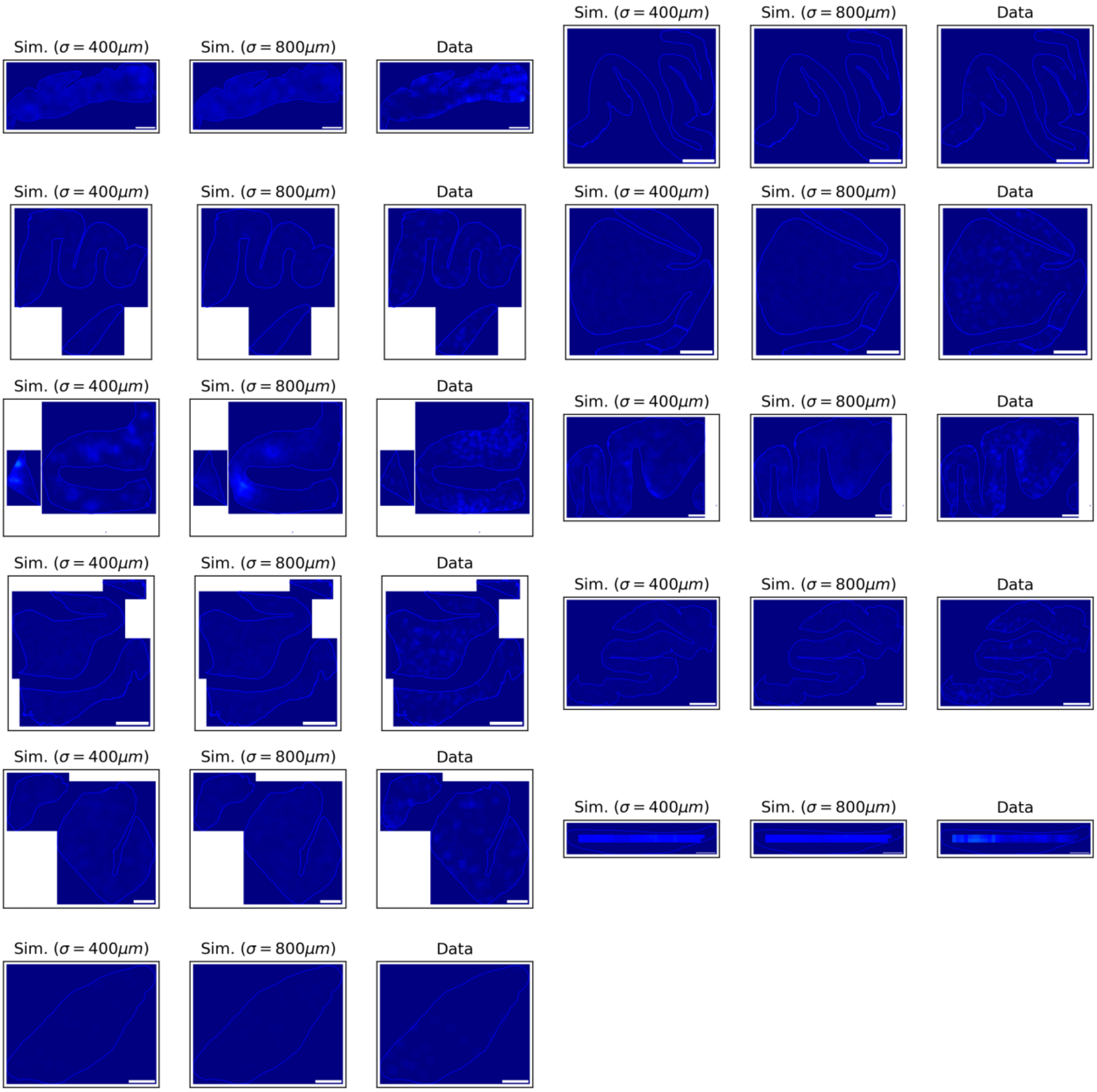


Figure S19: **Additional 2D image comparisons (Stage 4, patient ID = 6).** The simulation columns show the rolling average aggregate density from real data. The data columns show the corresponding simulations using the best-fit parameters. Scale bar = 2 mm.

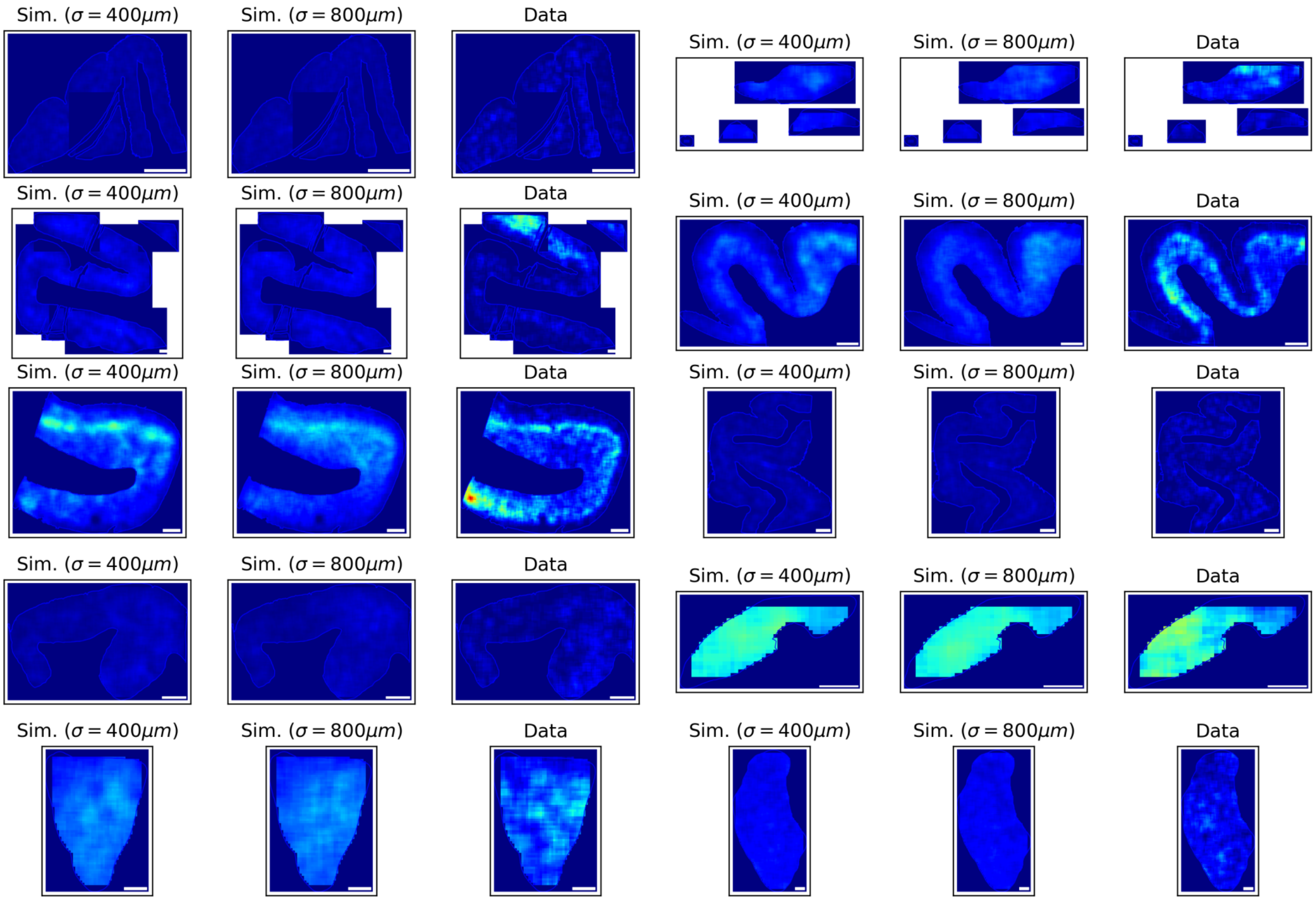


Figure S20: **Additional 2D image comparisons (Stage 5, patient ID = 7).** The simulation columns show the rolling average aggregate density from real data. The data columns show the corresponding simulations using the best-fit parameters. Scale bar = 2 mm.

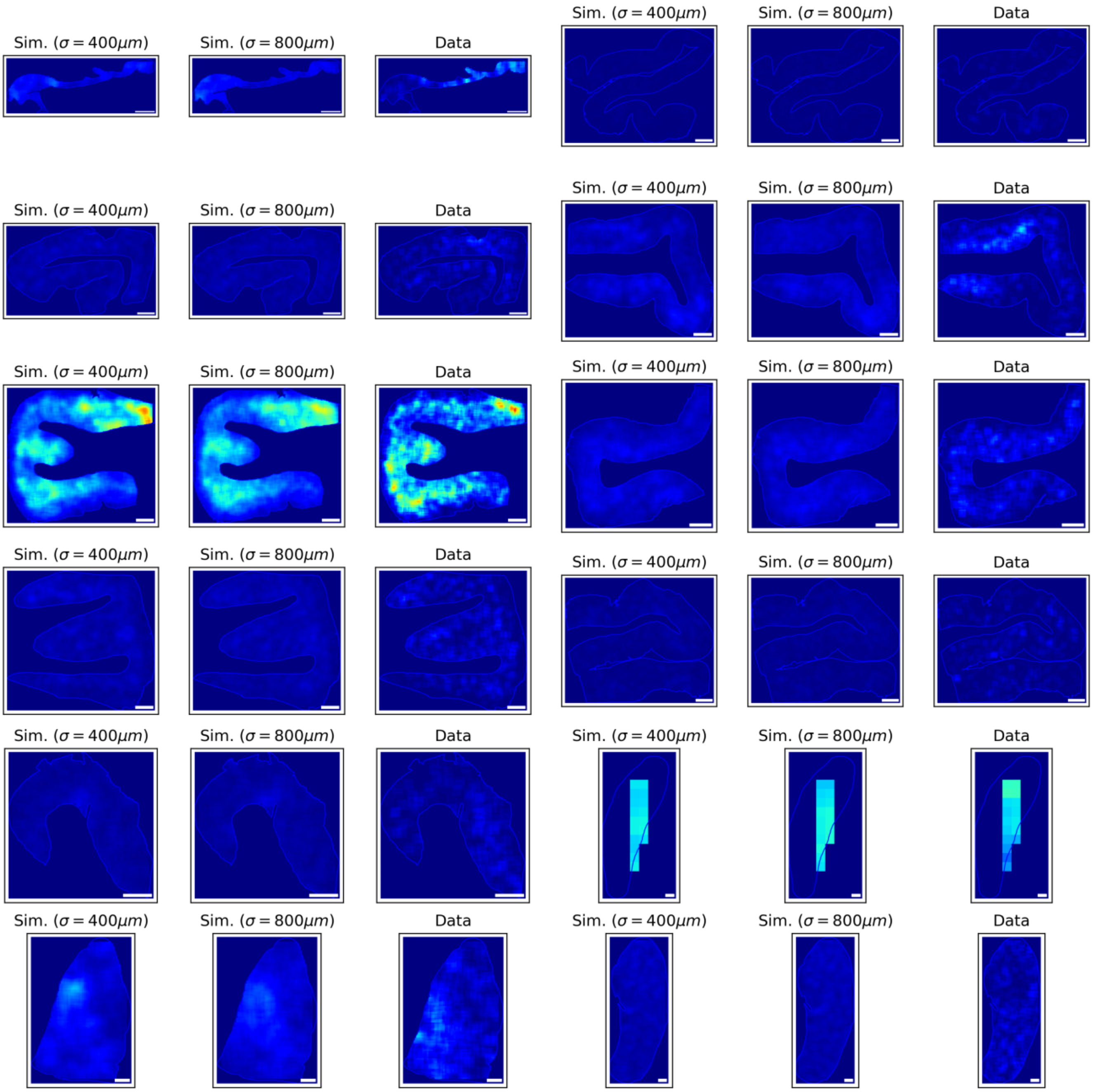


Figure S21: **Additional 2D image comparisons (Stage 5, patient ID = 8).** The simulation columns show the rolling average aggregate density from real data. The data columns show the corresponding simulations using the best-fit parameters. Scale bar = 2 mm.

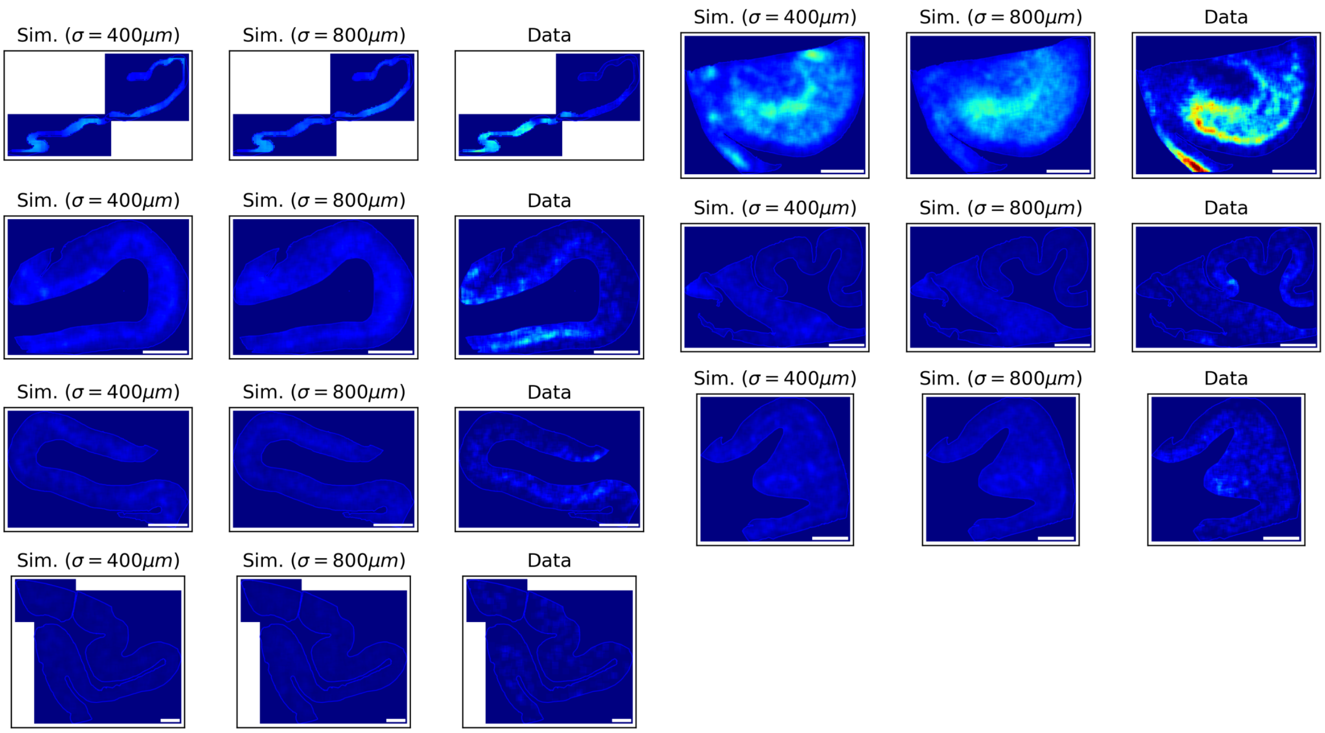


Figure S22: **Additional 2D image comparisons (Stage 5, patient ID = 9).** The simulation columns show the rolling average aggregate density from real data. The data columns show the corresponding simulations using the best-fit parameters. Scale bar = 2 mm.

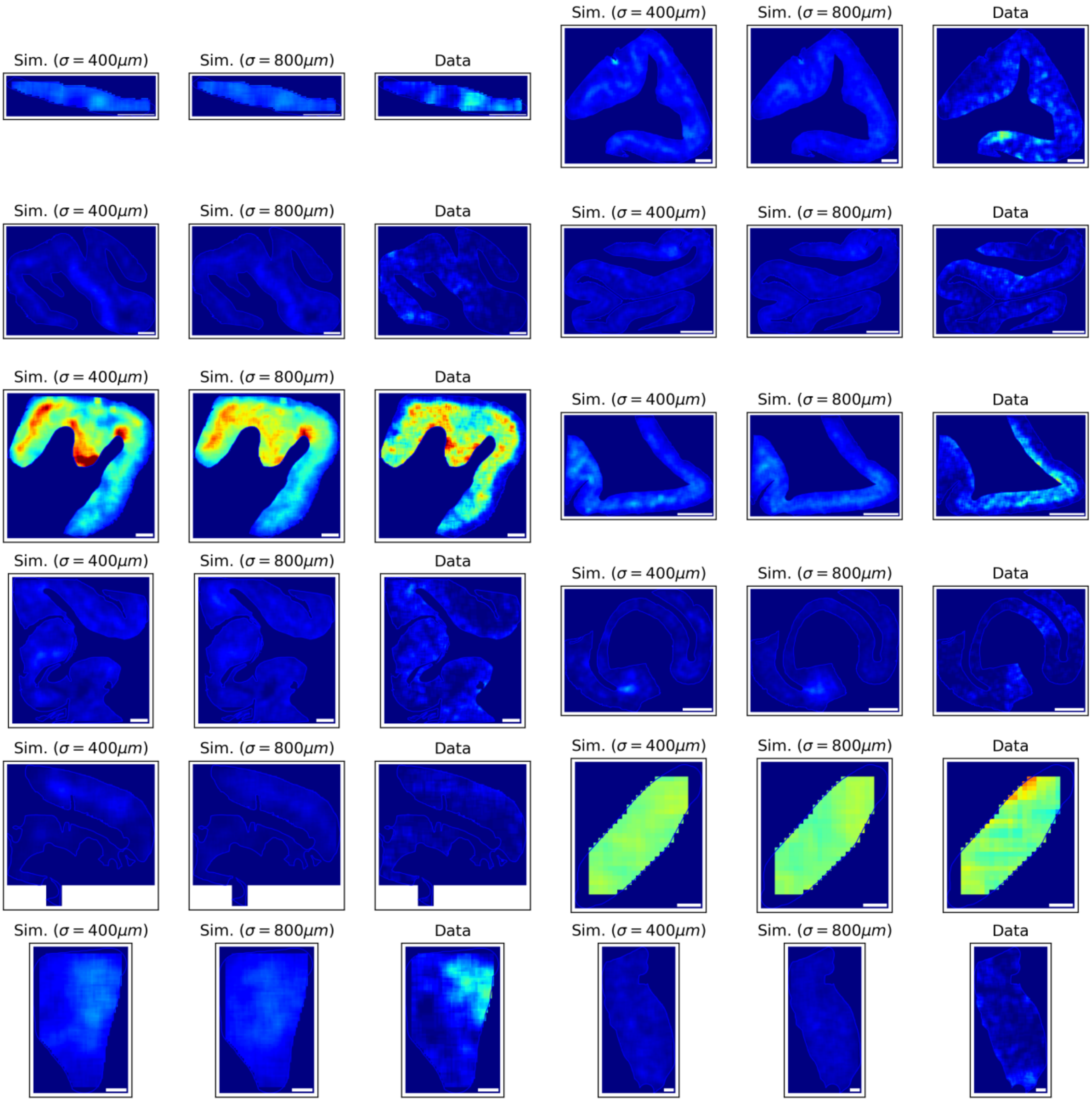


Figure S23: **Additional 2D image comparisons (Stage 6, patient ID = 10).** The simulation columns show the rolling average aggregate density from real data. The data columns show the corresponding simulations using the best-fit parameters. Scale bar = 2 mm.

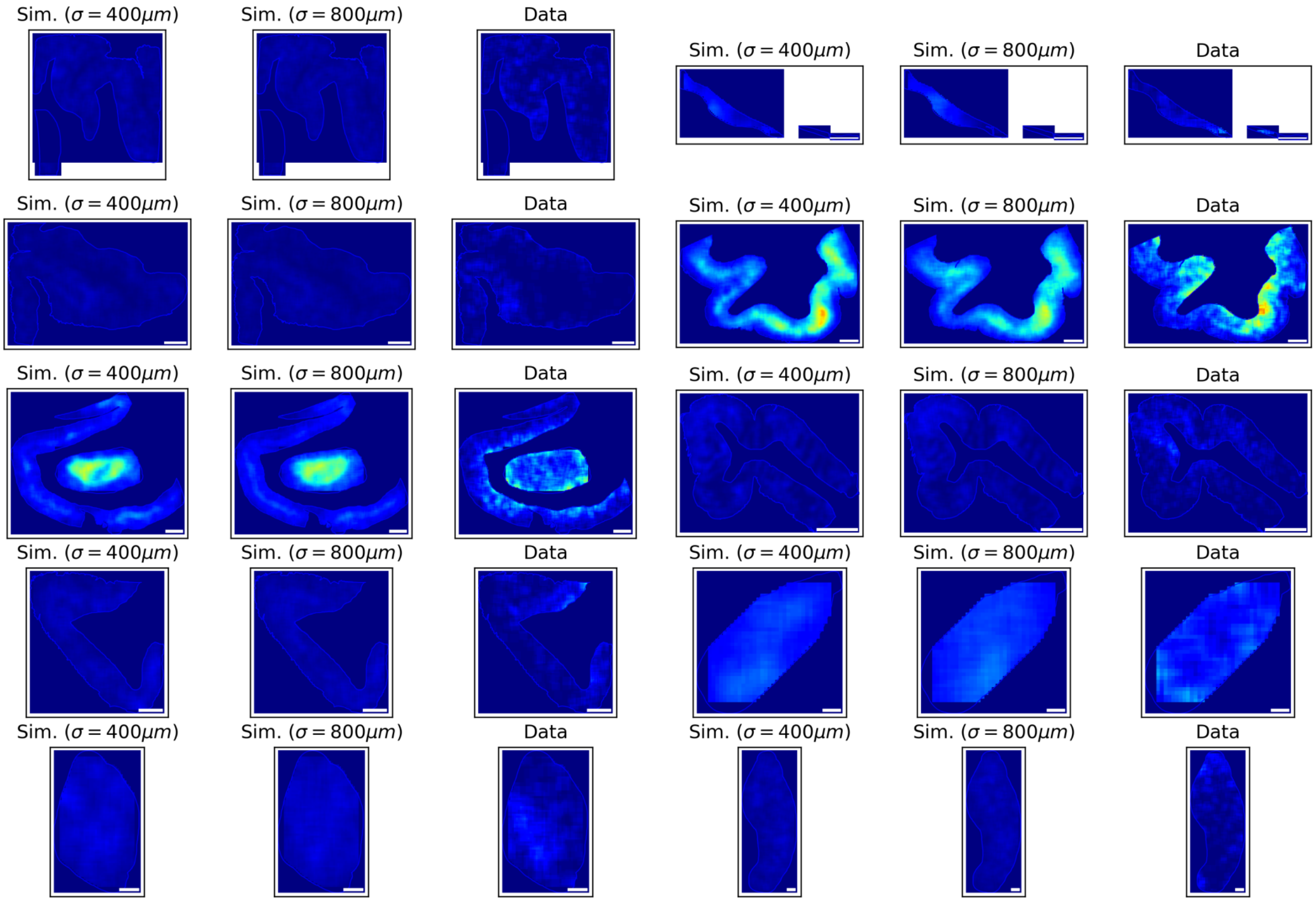


Figure S24: **Additional 2D image comparisons (Stage 6, patient ID = 11).** The simulation columns show the rolling average aggregate density from real data. The data columns show the corresponding simulations using the best-fit parameters. Scale bar = 2 mm.

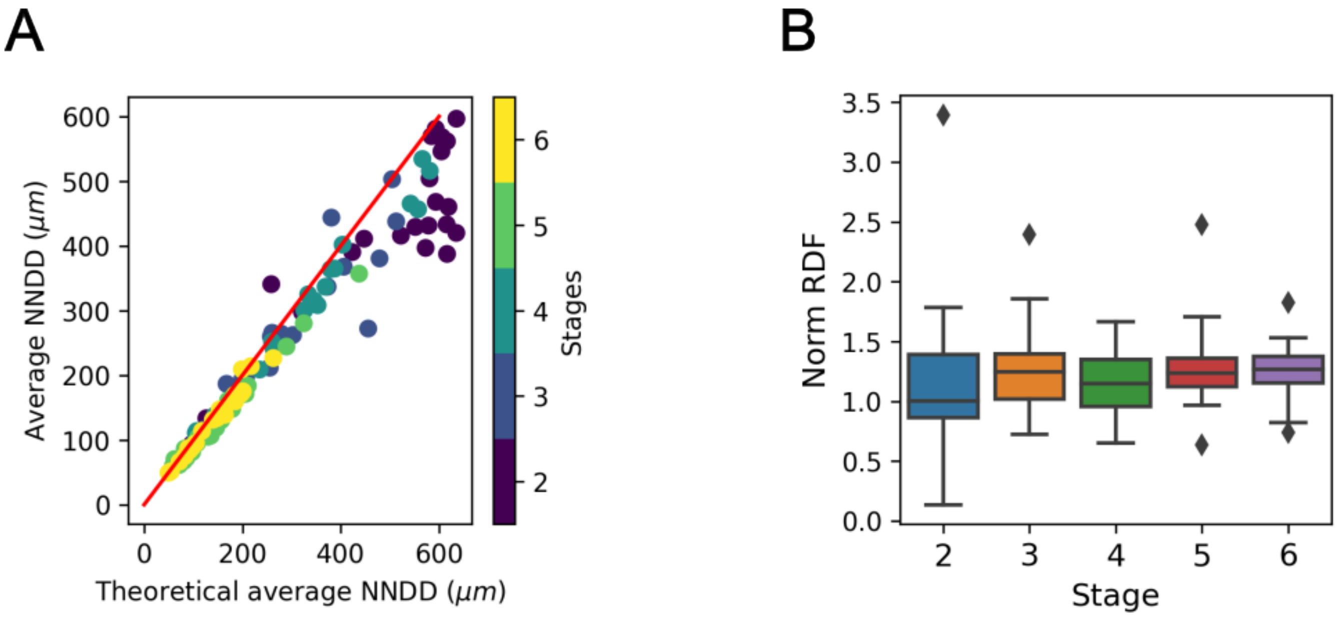


Figure S25: **More model-free analysis from the patient data.** (A) Average aggregate nearest neighbour distance determined in patient data, compared to that of a random distribution ($R^2 = 0.99$). The red curve represents the scenario where the measured values match the theoretical values. (B) Box plots showing the ratio between average value of the radial distribution function up to a distance of 1 mm and the average value of hypothetical radial distribution function up to a distance of 1 mm when aggregated cells are randomly distributed, grouped by stage. Values of the average RDF above 1 denote an increased clustering of aggregated cells within clusters of approximately 1 mm. This can be observed at all stages from stage 3 onward. In earlier stages, the number of aggregated cells is too low to draw clear conclusions. The box represents the interquartile range (IQR), encompassing the middle 50% of the data with edges at the first and third quartiles. Whiskers extend to 1.5 times the IQR from the quartiles to show the data range, while points outside these whiskers are plotted as outliers.

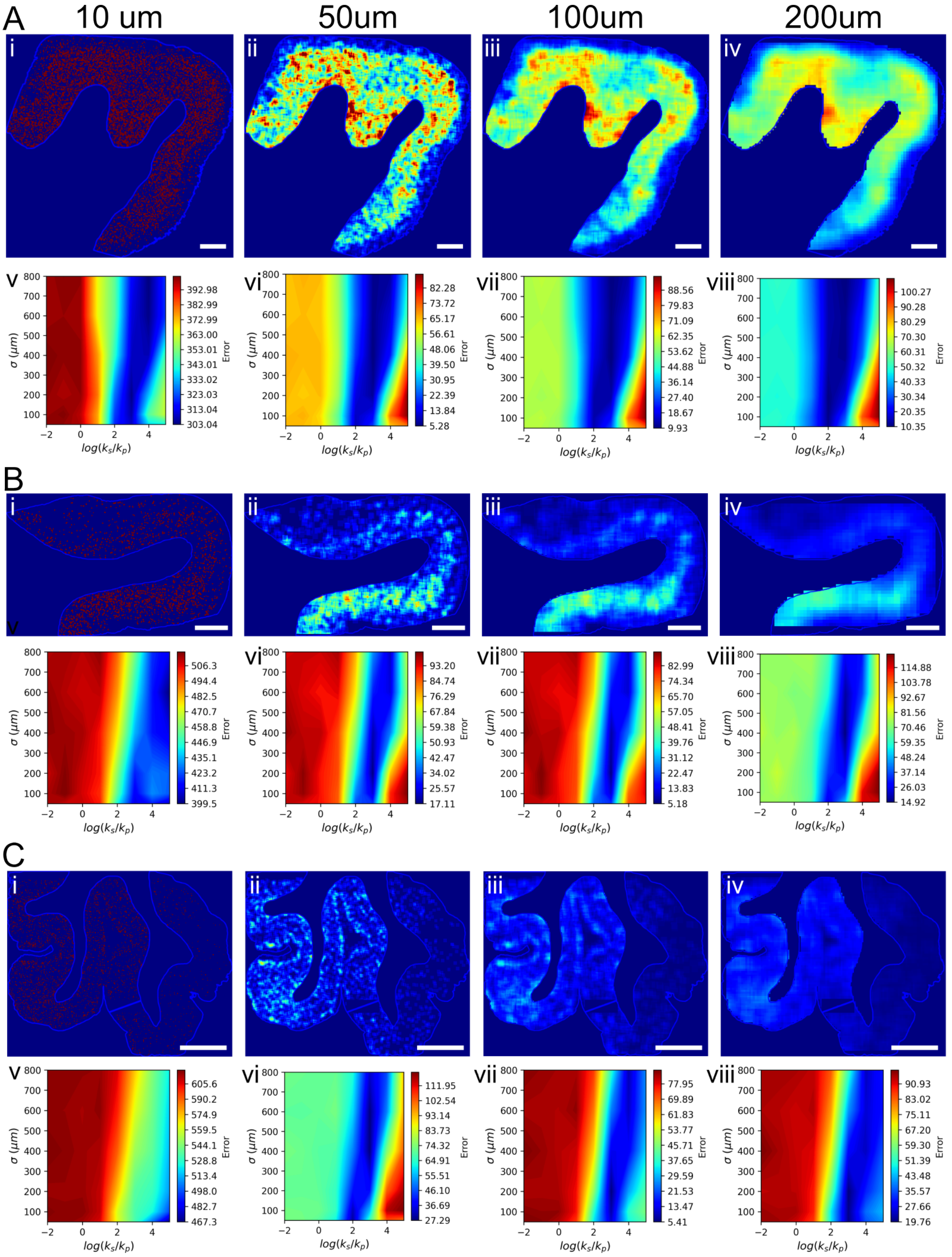


Figure S26: **Comparison of different sizes of rolling density window** (A)-(C) Varying rolling density window size from 10 $\mu m$ to 200 $\mu m$ from three example brain slices. In each panel, the top sub-panels show images of different window sizes. The bottom sub-panels shows the two-dimensional plots of the mean error across different parameter sets (see *Methods*) for the definition of error. Scale bar = 2 mm.

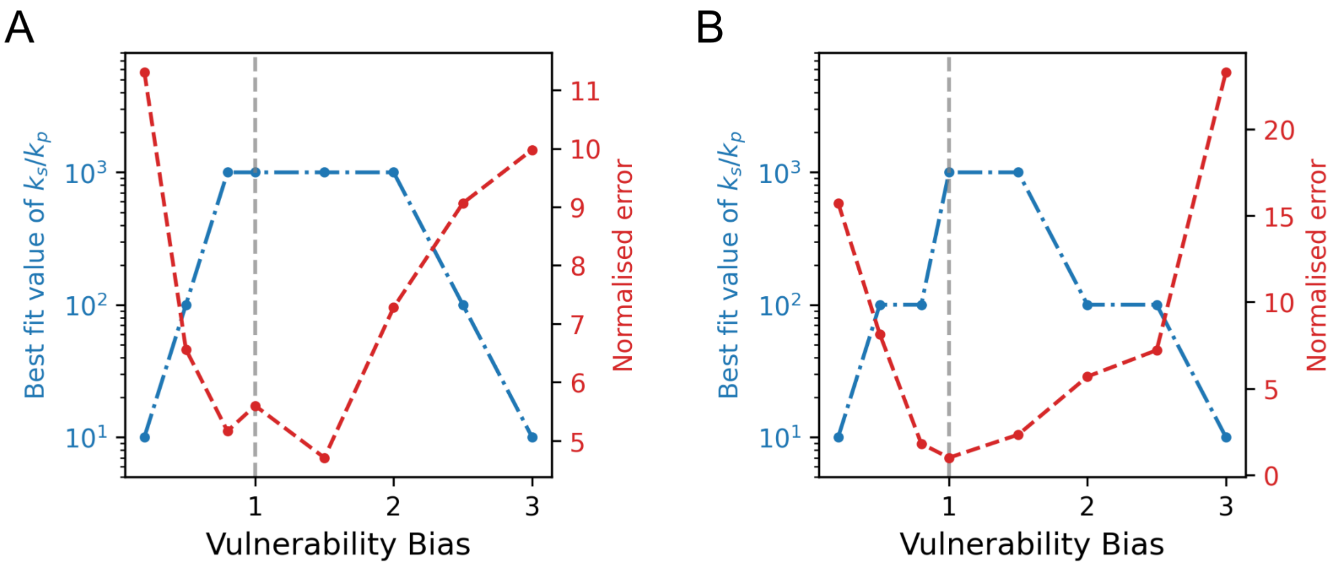


Figure S27: **Quantitative sensitivity analysis of the uniform vulnerability assumption.** (A) Results from the sensitivity analysis using the APN histogram for parameter inference. The plot shows the best-fit rate constant ratio ($k_s/k_a$, blue line, left axis) and the cost function ratio (red line, right axis) as a function of the vulnerability bias. (B) Results from the sensitivity analysis using the RDF for parameter inference, showing the same metrics as in (A). The vulnerability bias on the x-axis represents the fold-change in intrinsic vulnerability for cells in low-density regions, where a value of 1 corresponds to the uniform vulnerability model. The normalised error is a metric designed to test how well the parameters from the non-biased model can explain data generated under a biased reality. It is defined as the error between the patient data and the biased model, divided by the baseline error of the biased model fit to itself. For this analysis, the fixed "best-fit parameters from the non-biased model" were used: $k_s/k_a = 1000$, $\sigma = 400$ for APN histogram analysis and $k_s/k_a = 1000$, $\sigma = 800$ for RDF analysis.
